# Apnoea duration and changes in cardiorespiratory and cerebrovascular responses in preterm neonates: a systematic review and meta-analysis

**DOI:** 10.1038/s41390-025-04496-x

**Published:** 2025-10-28

**Authors:** Yiru Chen, Coen S. Zandvoort, Luke Baxter, Odunayo A. T. Fatunla, Vithushanan Ketheeswaranathan, Ravi Poorun, Zara Small, Fatima Usman, Matthew Henry, Luc Berthouze, Mauricio Villarroel, Caroline Hartley

**Affiliations:** 1 Department of Paediatrics, University of Oxford, Oxford, UK; 2 University of Exeter Medical School, University of Exeter, Exeter, UK; 3 Medical Sciences Division, University of Oxford, Oxford, UK; 4 Bodleian Health Care Libraries, University of Oxford, Oxford, UK; 5 Department of Informatics, University of Sussex, Brighton, UK; 6 Department of Engineering Science, University of Oxford, Oxford, UK

## Abstract

**Background:**

Apnoea is a common respiratory complication in preterm neonates, leading to substantial changes in physiology. We conducted this systematic review and meta-analysis to examine the relationship between apnoea duration and changes in heart rate, oxygen saturation, cerebral oxygenation and cerebral blood volume in preterm neonates, and to identify factors that modulate this relationship.

**Methods:**

We searched Medline, EMBASE, PsycINFO, and Cochrane Central Register of Controlled Trials databases and included primary empirical studies examining the relationship between apnoea or respiratory pause duration and at least one outcome in hospitalised neonates with postmenstrual age (PMA) <37 weeks. Through meta-analyses, we prospectively examined factors that may modulate this relationship, including postmenstrual age, medication use, and apnoea type.

**Results:**

Forty-two papers were included, involving a total of 1483 neonates with 2399 study sessions. The decrease in heart rate, oxygen saturation, and cerebral oxygenation were significantly correlated with apnoea duration. PMA significantly modulated the relationship, with younger neonates more likely to exhibit oxygen desaturation from short apnoeas.

**Conclusions:**

Cardiorespiratory and cerebrovascular responses to apnoea are correlated with apnoea duration, yet wide variability exists in the response. Further research is needed to identify how factors modulate the relationship.

**Impact:**

- Systematic review and meta-analysis of the relationship between apnoea duration and change in heart rate, oxygen saturation, cerebral oxygenation and cerebral blood volume in preterm neonates.
- Through meta-analysis, we demonstrate that postmenstrual age plays a significant modulating role in the relationship between apnoea duration and change in oxygen saturation, with younger infants more likely to have desaturations.
- Apnoea can lead to significant cardiorespiratory and cerebrovascular changes; understanding the factors which modulate these relationships are key to facilitating personalised alarm limits.

## Introduction

Globally, approximately 10% of neonates are born preterm (before 37 weeks of gestation).^1^ Apnoea, the cessation of breathing, is a common respiratory complication for these neonates, affecting more than 50%, especially those born extremely prematurely (before 28 weeks of gestation).^2^ Apnoea can occur multiple times per day and can lead to significant physiological changes, including alterations in heart rate, blood oxygen saturation, cerebral oxygenation, and cerebral blood volume.^3–6^ While most episodes resolve spontaneously or with minimal intervention, recurrent or prolonged episodes may have clinical significance and require appropriate monitoring and management. Additionally, apnoea has been linked to long-term complications, such as an increased incidence of retinopathy of prematurity (ROP)^7^ and cognitive deficits later in life.^8^ These may be related to the extent of apnoea-induced hypoxia or brain activity changes.^9^

There is wide variability within and between neonates in how they respond to an apnoea of a given duration, with short pauses in breathing sometimes leading to large changes in cardiorespiratory and cerebrovascular changes, and other times much longer pauses in breathing not leading to significant physiological instability.^4^ Factors such as the age of the neonate may alter cardiorespiratory and cerebrovascular responses to an apnoea, with older babies able to tolerate longer periods of apnoea without physiological instability. It also seems plausible that co-morbidities and medication have an impact. For example, caffeine reduces the incidence of apnoea and intermittent hypoxaemia.^10–12^ A better understanding of the factors which modulate the impact of apnoea on cardiorespiratory and cerebrovascular responses is crucial to identify neonates at the highest risk of physiological instability. Ultimately, this could lead to the development of predictive models that can guide treatment or facilitate closer monitoring for high-risk neonates.

We conducted this systematic review and meta-analysis to examine the current knowledge regarding the relationship between apnoea duration and physiological instability (specifically changes in heart rate, oxygen saturation, cerebral oxygenation, and cerebral blood flow) in preterm neonates, and to investigate factors which modulate these relationships. We aimed to investigate the following questions:

1. What are the relationships between the duration of pauses in breathing/apnoea and changes in heart rate, blood oxygen saturation, cerebral oxygenation, and cerebral blood volume in hospitalised premature neonates?
2. How do these relationships vary across neonates with different postmenstrual age (PMA), pathology (specifically sepsis and necrotising enterocolitis [NEC]), medication (specifically methylxanthines and opioids), and any other potential modulating factors identified in the included papers?

When writing the protocol, we additionally aimed to investigate the question ‘how are these relationships modulated by the frequency/clustering of pauses in breathing?’. However, no papers identified had data that enabled us to address this aim.

## Methods

This systematic review is reported in accordance with the Preferred Reporting Items for Systematic Reviews and Meta-Analyses (PRISMA) statement.^13^ The protocol for this systematic review was registered in PROSPERO (CRD42024534164).

### Eligibility criteria

All primary empirical and peer-reviewed studies reporting a relationship between apnoea or respiratory pause duration and at least one of the outcomes (change in heart rate, blood oxygen saturation, cerebral oxygenation and cerebral blood volume) in hospitalised human neonates with PMA < 37 weeks were included. We excluded papers with the wrong population (non-human species; pre-natal humans; full-term neonates (PMA ≥ 37 weeks) and older (e.g., children, and adult subjects); neonates with neurological abnormalities (e.g. seizures) or congenital abnormalities; and non-hospitalised neonates. We also excluded papers with the incorrect study characteristics (secondary literature (e.g. reviews, book chapters); non-empirical literature (e.g. opinions, commentaries, perspectives); non-peer-reviewed grey literature (e.g. conference abstracts, meeting reports, theses); and study protocols). There were no restrictions based on publication language or date.

For studies that included neonates with both PMA < 37 weeks and ≥37 weeks, inclusion of the study was permitted. Where possible, data from neonates with PMA ≥ 37 weeks were excluded from the analysis.

### Search strategy

A combination of subject headings terms and controlled keywords were used, and searches were conducted initially on 23 November 2023 and updated on 10 December 2024 in the following databases: MEDLINE (Ovid), Embase (Ovid), PsycINFO (Ovid) and Cochrane Central Register of Controlled Trials (Cochrane Library, Wiley). Detailed searching strategies are included in Supplementary Methods. Additionally, backward citation tracking was carried out on the final set of papers as an additional information source, and this was done through the Citationchaser package.^14^

### Selection process

A systematic review management tool, Covidence (Melbourne, Australia), was used to manage records and data throughout the review. The references were imported onto the platform, and automatic de-duplication was performed. In total, seven reviewers were involved in the selection process. The selection followed a two-stage process:

1. A title and abstract screening against the inclusion criteria to identify potentially relevant papers.
2. A full-text screening of all the papers identified as possibly relevant for inclusion from the initial screening above.

At the title and abstract screening stage, a pilot block of 20 randomly selected articles was screened by all reviewers to ensure consistency of reviewers across the screening process. Papers were then divided into blocks, with two independent reviewers assigned to each block. Papers whose abstract did not describe any form of relationship between apnoea and changes in vital signs were excluded. At the full-text screening stage, the same two reviewers assessed all papers, except those written in non-English languages. Papers written in Dutch, German, Italian, or French were reviewed by fluent speakers of the respective language, with an additional reviewer using Google Translate to validate the results. One paper in Bulgarian and one in Danish were screened using Google Translate only and excluded at the title and abstract screening stage. At both stages, screening was carried out in duplicate and independently by the two reviewers, and inconsistencies were settled by discussion between both reviewers and if necessary, an arbitrator. For consistency, one reviewer (YC) acted across all papers. The same arbitrator (LBa) also acted across all papers.

### Data collection process and data items

Data extraction was carried out by a single reviewer (YC). When relevant information was provided only in figure format, the PlotDigitizer software was used to extract values from the figures. This process was conducted twice for each figure to ensure consistency in the results.

The following variables were extracted: author’s names; year of publication; country and region of publication (according to World Health Organisation categorisation^15^); hospital(s) where data was collected, study period; study design; sample size; neonate’s age (gestational age and postmenstrual age), including median and range where available; type of respiratory support (the use of mechanical or non-mechanical devices to assist or maintain breathing, such as CPAP, NIPPV, mechanical ventilation, high-flow nasal cannula, and other forms of ventilator support) received by the neonates (if any); supplemental oxygen (the administration of additional oxygen, regardless of whether breathing support is used) received by the neonates (if any); the pathological conditions of the neonates (specifically sepsis and NEC); type of medication used (if any); description of apnoea/pause in breathing (i.e., duration, method of measurement, any resuscitation provided, information on the frequency of pause/clustering of pauses); description of the changes in outcome variables (i.e., absolute change, baseline and minimum/maximum values if provided, method of measurement); duration of the recordings and time of day; signal quality measures (if given). We also actively sought evidence for any other potential modulators of the relationship between apnoea duration and physiological instability investigated within the studies included in the review.

### Study risk of bias assessment (quality assessment)

Assessment of the risk of bias in the included studies was carried out by using the checklist for systematic reviews and research syntheses provided by the Joanna Briggs Institute Critical Appraisal tools. Two reviewers (Y.C. and C.Z.) evaluated the risk of bias independently. Any disagreement between the two reviewers was settled through discussion.

To derive an overall risk of bias rating,^16,17^ we focused exclusively on items within the JBI checklists related to internal validity. Each item in the relevant JBI checklists was classified as either “critical” (pertaining to internal validity) or “non-critical” (pertaining to other constructs). Critical items were mapped to established domains of bias, including selection bias, confounding, measurement bias, temporal bias, and attrition bias. Studies were then rated based on their responses to critical items, following a predefined rule set: low risk of bias was defined as no “no” responses on critical items; moderate risk of bias was defined as one “no” response on a critical item; high risk of bias was defined as two or more “no” responses on critical items. Responses of “unclear” or “not applicable” were not treated as indicators of bias, ensuring a conservative and transparent assessment approach.

### Data synthesis

The outcome of the database searches and study selection process was presented in a PRISMA flowchart. The data synthesis was performed using a comprehensive narrative approach to ensure a rigorous and informative analysis. Narrative and qualitative synthesis involved the tabulation of results where feasible.

Quantitative data from the selected studies regarding the relationship between apnoea duration and cardiorespiratory and cerebrovascular responses in preterm neonates were extracted and pooled for meta-analysis where available. The dependent variable was the mean percentage changes in cardiorespiratory/cerebrovascular response. If a study provided the values for percentage change in cardiorespiratory/cerebrovascular response directly, these were used for the meta-analysis. When values at the beginning and end of apnoea were provided, the difference was calculated and divided by the starting value to determine the percentage change. The independent variable was the apnoea durations. If a study provided the mean apnoea durations for each pooled point, these were used directly. When only a range of apnoea durations was provided, the midpoint of the range was used as the X-value (e.g., if the range of apnoea duration was 10–20 s, the X-value would be 15 s for that point). If multiple studies presented the same results using the same dataset, then we only included the results once in the meta-analysis.

Pooled data were analysed using Pearson correlation coefficients, linear regression models, and linear mixed-effects models. For the linear regression models, apnoea duration was the predictor, and changes in cardiorespiratory/cerebrovascular variable were the response. The square root of the number of apnoea events associated with each data point was used as the weight in the model. The coefficient of determination (*R*^2^) was used to assess how much of the variation in vital signs could be explained by apnoea duration. The formula to calculate *R*^2^ was:

$${R}^{2}=1-\,\frac{S{S}_{{RES}}}{S{S}_{{tot}}},$$

where $S{S}_{{RES}}$ is the sum of squares of residuals, and $S{S}_{{tot}}$ is the total sum of squares.

For the linear mixed-effects models, the study ID number was used as a random effect, and apnoea duration as a fixed effect. The impact of apnoea type (whether the reported apnoea is central, mixed, obstructive, or a combination [i.e. the study authors combined types within their analysis]), PMA, and the use of theophylline (a medication that is commonly used for apnoea treatment) on the relationship between apnoea duration and cardiorespiratory/cerebrovascular response was analysed, where applicable, by adding interaction terms between these factors and apnoea duration as fixed effects in the model.

For a post hoc analysis of how PMA affects the relationship between changes in oxygen saturation (SpO2) and apnoea duration, we used a linear regression model with the formula:

$${{{\rm{change}}}}\; {{{\rm{in}}}}\; {{{\rm{SpO}}}}2\,= {{{{\rm{\beta }}}}}_{0}+{{{{\rm{\beta }}}}}_{1}\cdot {{{\rm{Duration}}}}+{{{{\rm{\beta }}}}}_{2}\cdot {{{\rm{PMA}}}}\\ +{{{{\rm{\beta }}}}}_{3}\cdot ({{{\rm{Duration}}}}\times {{{\rm{PMA}}}})+{{{\rm{\varepsilon }}}}$$

Using this model, we predicted the apnoea duration at which oxygen saturation is likely to drop by more than 10% for neonates of different PMAs. The 10% threshold is commonly used for defining neonatal desaturation.^18^

The correlation coefficients from different papers reporting the same outcome were standardised and converted into a common effect size metric, Fisher’s Z score, which was combined to provide overall correlation factors. The regression lines from the individual studies were plotted alongside the line obtained from the meta-analysis for visual comparison of the variation across studies.

## Results

**Fig. 1:**
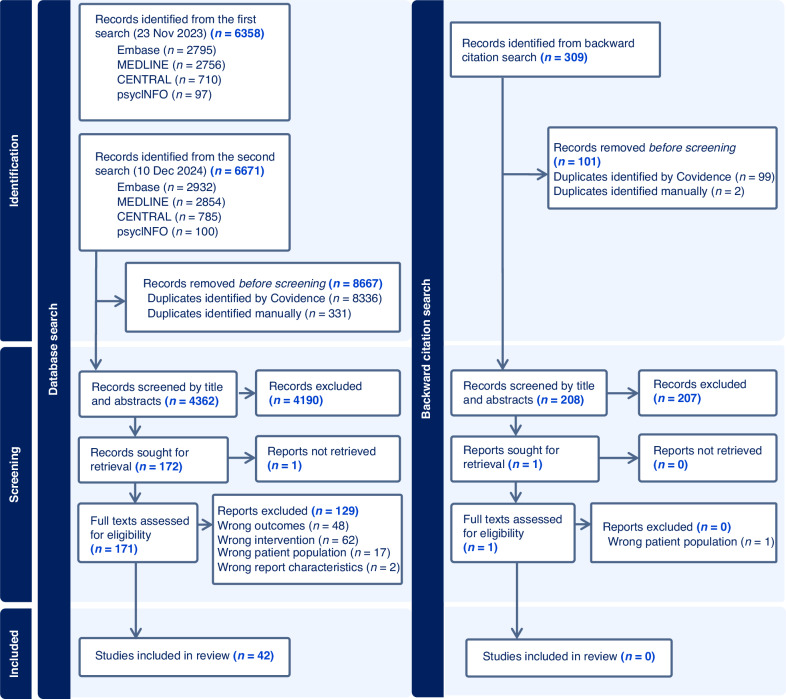
Prisma flow chart showing the study selection process.

A total of 4362 studies were identified, after the removal of 8567 duplicates (Fig. 1). Following the title and abstract screening, 4190 studies were excluded, and a further 129 studies were excluded after the full-text review. One study^19^ was excluded due to unavailability of the full text after database searches and inter-library requests. Overall, 42 studies were included in this review, the summarised details of the included studies can be found in Table 1, and the detailed extracted data are provided in Supplementary Table S1.

**Table 1:** Summarised details for all included studies.

Characteristic	Value
Total number of studies	42
Total number of neonates	1482
Total number of recording sessions	2399
Number of neonates per study	Median 24, range: 1–335, IQR: 14–31.75
Number of sessions per study	Median 31, range: 1–386, IQR: 18–78
Study design	23 cross-sectional (54.8%) 16 cohort (38.1%) 2 case-control (4.8%) 1 case report (2.4%)
Publication year	Median 1992, range 1969–2022
Geographic region (WHO classification)	21 from Europe (50%), 17 from Americas (40.5%), 4 from Western Pacific (9.5%), 0 from SE Asia, Eastern Mediterranean, or Africa

The included studies reported the relationship between apnoea duration and one or more physiological outcome measures: change in heart rate (*n* = 32, 76.2%), change in oxygen saturation (*n* = 25, 60.0%), change in cerebral oxygenation (*n* = 2, 4.8%), and change in cerebral blood volume (*n* = 3, 7.1%). Details of the studies reporting each outcome are presented in Table 2.

**Table 2:** Demographic details for the studies associated with each outcome measures. #: Some of these studies did not specify the exact number of neonates receiving the relevant treatment.

Outcome Measure	Heart rate	Oxygen Saturation	Cerebral Oxygenation	Cerebral Blood Volume
Number of studies	32	25	2	3
Number of patients	1044	1122	97	114
Number of recording sessions	>1792 (one study only stated that multiple recording was available for each infant, without stating the exact number)	1875	458	475
Postmenstrual age (PMA) at study [weeks]	10 studies: averaged mean PMA = 35.0 weeks (237 neonates). 10 studies: PMA reported in alternative metrics such as median or range. 12 studies: PMA not specified numerically.	13 studies: averaged mean PMA = 35.6 weeks (378 neonates). 7 studies: PMA reported in alternative metrics such as median or range. 5 studies: PMA not specified numerically.	1 study: averaged mean PMA = 34.2 weeks (39 neonates). 1 study: PMA not specified numerically.	2 studies: averaged mean PMA = 34.9 weeks (56 neonates). 1 study: PMA not specified numerically.
Respiratory stimulants at the time of study	8 studies: no respiratory stimulant. 15 studies: at least 102 neonates # received respiratory stimulants. 9 studies: not specified.	7 studies: no respiratory stimulant. 14 studies: at least 130 neonates # received respiratory stimulants. 4 studies: not specified.	2 studies: 37 infants received respiratory stimulants.	3 studies: 47 infants received respiratory stimulants.
Respiratory support at the time of study	10 studies: no respiratory support. 7 studies: 287 neonates receiving respiratory support. 15 studies: not specified.	7 studies: no respiratory support. 7 studies: at least 320 neonates # receiving respiratory support at the time of study. 11 studies: not specified.	1 study: no respiratory support. 1 study: not specified.	1 study: no respiratory support. 2 studies: not specified.
Supplemental oxygen at the time of study	12 studies: no supplemental oxygen. 6 studies: at least 75 neonates# received supplemental oxygen. 14 studies: not specified.	11 studies: no supplemental oxygen. 5 studies: at least 42 neonates # received supplemental oxygen. 9 studies: not specified.	1 study: no supplemental oxygen (58 infants). 1 study: not specified.	1 study: no supplemental oxygen (58 infants). 1 study: 9 infants received supplemental oxygen. 1 study: not specified.
Measuring techniques	ECG (*n* = 26). Plethysmography (*n* = 4). Impedance (*n *= 6). Thermistor (*n* = 3).	Pulse oximetry (*n* = 25)	NIRS (*n* = 2)	NIRS (*n* = 3)
Metrics used	Relative change in HR (*n* = 6). Absolute change in HR (*n* = 3). Minimum HR value (*n* = 1). Bradycardia incidences (*n* = 6). Correlation coefficients (*n* = 6). Linear regression results (*n *= 2). Other metrics only (*n* = 8.	Relative change in SpO2 (*n *= 5). Minimum SpO2 (*n* = 1). Desaturation instances (*n *= 2). Correlation coefficients (*n* = 9). Linear regression (*n* = 3). Duration of apnoea with and without desaturation (*n* = 2). Other metrics only (*n* = 7).	Change in cerebral Hb difference (cHbD) (*n* = 2).	Change in total cerebral blood volume (CBV) (*n* = 1). Amplitude of total Hb (tHb) (*n* = 1). Change in concentration changes of total cerebral haemoglobin (cHbtot) (*n* = 1).

The definition of apnoea, desaturation, and bradycardia events investigated in the different studies varied as summarised in Table 3. The techniques used to measure apnoea also varied, including polysomnography, acoustic monitoring, inductive plethysmography, thermistor, and pneumotachograph (Supplementary Tables S2–S5).

**Table 3:** Definition of apnoea, desaturation and bradycardia used across the studies.

Parameter	Variations
Definition of apnoea	Cessation of breathing longer than: ≥3–5 s (*n *= 6 studies); ≥10 s (*n* = 28 studies); ≥15–20 s (*n* = 7 studies). 9 studies required associated desaturation or bradycardia to define apnoea.
Definition of desaturation	Oxygen saturation <90% in 7 studies, <85% in 4 studies, and <80% in 6 studies. 5 studies defined desaturation as a ≥ 10% drop from baseline.
Definition of bradycardia	Heart rate <90 bpm (*n* = 5 studies), or <100 bpm (*n* = 15 studies). 3 studies defined bradycardia as a ≥20% drop from baseline. 3 studies graded severity (mild vs. severe).

### Relationship between apnoea duration and change in heart rate

A total of 32 articles investigated the change in heart rate compared with apnoea duration (Supplementary Table S1), involving 1008 neonates and 1734 recording sessions in total. Most studies (*n* = 26, 83.9%) measured heart rate using the electrocardiogram (ECG), while some used different monitors (Table 2). The relationship between heart rate changes and apnoea duration was examined using various methods, including correlation coefficients, linear regression models, and scatter plot diagrams (Table 4). Most studies demonstrated that a longer apnoea duration is correlated with a greater reduction in heart rate (Table 4).

**Table 4:** Summarised relationships between apnoea duration and change in heart rate.

Metric	Relationship with apnoea duration
Relative change in heart rate (*n* = 6 studies)	A meta-analysis was conducted on all six studies but did not find a significant relationship (slope = −0.27, *R*^2^ = -0.05; *r* = −0.31, *p* = 0.062). After removing one study with different apnoea definition [required bradycardia], the relationship became significant (slope = −1.55, *R*^2^ = 0.36; *r* = −0.61, *p* = 0.0019).
Absolute change in heart rate (*n* = 3 studies)	All three studies found that longer apnoea durations were associated with greater reductions in heart rate during the events. However, as the original data points and correlation coefficients were not available, the results could not be pooled for meta-analysis.
Minimum heart rate value (*n* = 1 study)	Central apnoea was the shortest in duration and had the highest minimum heart rate value during apnoea. Mixed apnoea had the longest duration and the lowest minimum heart rate value during apnoea.
Bradycardia incidences (*n* = 6 studies)	A meta-analysis was conducted on all six studies but did not find a significant relationship (slope = 1.71, *R*^2^ = −0.05; *r* = 0.44, *p* = 0.062). After removing one study with different apnoea definition [required bradycardia], the relationship became significant (slope = 2.31, *R*^2^ = 0.57; *r* = 0.81, *p* < 0.0001).
Correlation coefficients (*n* = 6 studies)	The correlation coefficients were calculated based on different heart rate metrics: Minimum heart rate: *n* = 2 studies (magnitude of r ranged between 0.10 and 0.77), Relative change in heart rate: *n* = 1 studies (*r* = 0.09, *p* = 0.001), Bradycardia incidence: *n* = 1 study (magnitude of *r* for different apnoea types ranged between 0.51 and 0.65), Area under the heart rate curve: *n* = 1 study (*r* = 0.81, *p* < 0.001), Time of onset of bradycardia: *n* = 1 study (*r* = 0.73, *p* < 0.0001).
Linear regression results (*n* = 2 studies)	One study showed that minimal heart rate=74.22-0.14*duration of apnoea (*r* = 0.11, *p* < 0.0001). The other study modelled the decrease in heart rate as a function of apnoea duration and showed an average accuracy of 73%.
Other metrics (*n* = 11 studies)	Eleven studies did not include any of the specific metrics described above. 10 of these studies reported significant relationships between apnoea duration and changes in heart rate (metrics varied). One study reported that longer apnoea did not cause more drops in heart rate.

**Fig. 2:**
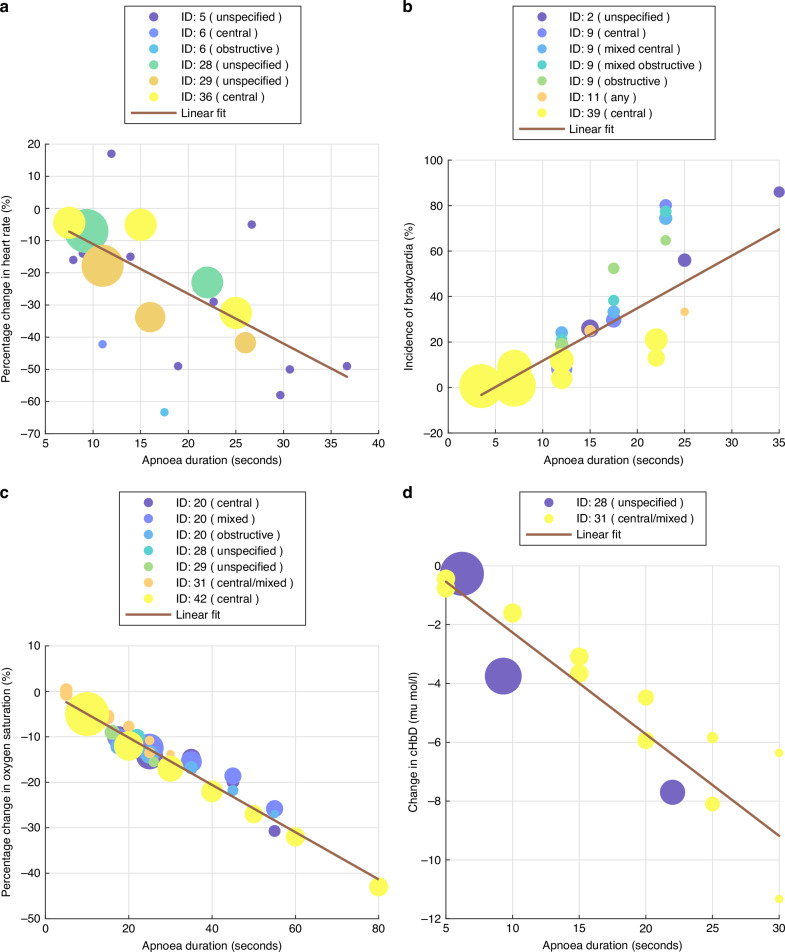
Meta-analysis of change in physiology compared with apnoea duration. **a** The percentage change in heart rate during apnoea compared with apnoea duration. **b** The percentage of apnoea events accompanied by bradycardia against apnoea duration. **c** The percentage change in oxygen saturation during apnoea compared with apnoea duration. **d** The change in cerebral haemoglobin difference (cHbD) during apnoea against apnoea duration. The size of the points indicates their weight in producing the linear best fit, calculated as the square root of the number of apnoea episodes associated with each point. ID: 2 – Gabriel et al.,^26^ ID: 5 – Fenichel et al.,^20^ ID: 6 – Vyas et al.,^24^ ID: 9 - Henderson-Smart et al.,^28^ ID: 11 – Mathew,^29^ ID: 20 - Finer et al.,^21^ ID: 28 – Urlesberger et al.,^25^ ID: 29 – Carbone et al.,^22^ ID: 31 – Pichler et al.,^5^ ID: 36 – Beck et al.,^23^ ID: 39– Marshall et al.,^30^ ID: 42 – Varisco et al.^52^

Six studies^20–25^ presented the mean change in heart rate during apnoea for different apnoea duration subgroups or reported the information where the mean change can be inferred. These studies were included in the meta-analysis. The average of the mean PMA of the neonates across the six studies was 32.84 ± 1.56 (mean ± SD). The pooled data across all six studies did not show a significant relationship between apnoea duration and heart rate change (Supplementary Fig. S1, *n* = 6 studies; slope = −0.27, *R*^2^ = −0.05; *r* = −0.31, *p* = 0.062). However, data from Finer et al.^21^ showed an opposite trend to the other four studies, demonstrating a smaller change in heart rate for longer apnoeas. This might be due to the study’s unique definition of apnoea—the authors only recorded apnoeas associated with bradycardia or hypoxaemia, excluding shorter pauses with less significant decreases in heart rate. Removing this study from further meta-analysis, the pooled data from the other four studies showed a negative relationship between apnoea duration and percentage change in heart rate during apnoea (Fig. 2a, *n* = 5 studies; slope = −1.55, *R*^2^ = 0.36; *r* = −0.61, *p* = 0.0019).

Six studies^21,26–30^ reported the percentage of apnoea episodes associated with a bradycardia event. Gabriel et al.^27^ was not included in the meta-analysis as the data and results were the same as Gabriel et al.^26^ The remaining five studies^21,26,28–30^ were pooled in a separate meta-analysis; four of them^21,26,29,30^ used heart rate below 100 bpm as the definition of bradycardia whilst Henderson-Smart et al.^28^ defined bradycardia as a fall in heart rate of more than 30% below the baseline before the apnoea event. Generally, longer apnoeas were associated with higher rates of bradycardia (Supplementary Fig. S2, *n* = 5 studies; slope = 1.71, *R*^2^ = -0.05; *r *= 0.44, *p* = 0.0052). The study from Finer et al.^21^ was again removed from the meta-analysis due to its unique definition of apnoea. The pooled data from the other four studies showed a positive correlation between apnoea duration and the percentage of apnoeas associated with bradycardia (Fig. 2b, *n* = 4 studies, slope = 2.31, *R*^2^ = 0.57; *r* = 0.81, *p* < 0.0001).

There were 21 other studies which used a variety of heart rate metrics (e.g. absolute heart rate values during apnoea and multivariate linear analysis outcomes assessing the dependence of normalised percentage changes in heart rate on apnoea duration) and could not be pooled for additional meta-analysis due to limited data availability for each metric^3–5,18,31–47^ (Supplementary Table S1, Supplementary Table S2). The majority (*n* = 20 studies) demonstrated a statistically significant relationship between change in heart rate and apnoea duration (Table 4). In contrast, Curzi-Dascalova et al.^43^ studied 602 pauses in total, all lasting less than 12 s. Pauses of 10 to 12 s were rare (11 detected from all eligible babies) and did not induce higher cardiac deceleration than shorter pauses.

### Relationship between apnoea duration and change in oxygen saturation

A total of 25 articles investigated the relationship between change in oxygen saturation with apnoea duration (Supplementary Table S3), involving 1199 neonates and 1970 recording sessions in total. All the studies used a pulse oximeter to measure oxygen saturation.

As with the studies investigating changes in heart rate, a wide variety of oxygen saturation metrics and statistical analysis techniques were used (Table 5). Nine studies^4,21,31,44,45,48–51^ reported correlations coefficients between apnoea duration and different oxygen saturation metrics (change or drop in oxygen saturation: *n* = 4 studies, min oxygen saturation: *n* = 2 studies, duration of desaturations: *n* = 1 study, rate of desaturations: *n* = 1 study, area under the oxygen saturation curve: *n* = 1 study). Seven of these studies^4,21,31,44,45,48,49^ reported significant correlation coefficients, all indicating that longer apnoea durations were associated with greater decreases in oxygen saturation or longer desaturations. In contrast, Poets et al.^50^ reported no significant correlation between the duration of the apnoeic pause and the duration of desaturation (*r* = 0.3; *P* > 0.05, Spearman’s rank correlation test). Similarly, Adams et al.^51^ found no significant correlation between the length of respiratory pauses and the rate of desaturations (*r* = −0.20). Both of these studies recorded all desaturation or hypoxaemic events and then analysed the apnoea episodes associated with them, whereas other studies conducted the analysis in the opposite direction—starting with apnoea episodes and examining the associated desaturation events. The 4 studies^4,21,31,48^ that reported correlation coefficients based on the percentage decrease in oxygen saturation were pooled together. The pooled correlation was 0.37 (*n* = 4 studies, *p* < 0.0001, 95% confidence interval: [0.34, 0.39]).

**Table 5:** Summarised relationships between apnoea duration and change in oxygen saturation.

Metric	Relationship with apnoea duration
Relative change in oxygen saturation (*n* = 5 studies)	Meta analysis demonstrated a significant relationship (slope = −0.52, *R*^2^ = 0.95; *r* = −0.98, *p* < 0.0001).
Minimum oxygen saturation value (*n* = 1 study)	Oxygen saturation fell more during longer apnoea episodes.
Desaturation incidences (*n* = 2 studies)	One study found that the longest duration of desaturation <80%, and total duration of desaturation <90% were greater in prolonged apnoea comparing to shorter apnoea. The other study reported the percentage of apnoea episodes associated with desaturations according to apnoea type and corresponding duration ranges. However, the use of duration ranges prevented the data from being pooled.
Correlation coefficients (*n* = 9 studies)	The correlation coefficients were calculated based on different oxygen saturation metrics: Change or drop in oxygen saturation: *n* = 4 studies (pooled *r* = 0.37, *p* < 0.0001, 95% confidence interval: [0.34, 0.39]), Minimum oxygen saturation: *n* = 2 studies (magnitude of r for different groups ranged between 0.44 and 0.67), Duration of desaturations: *n* = 1 study (*r* = 0.3; *P* > 0.05), Rate of desaturations: *n* = 1 study (not significant, *r* = −0.198), Area under the oxygen saturation curve: *n* = 1 study (*r* = 0.88, *p* < 0.001).
Linear regression results (*n* = 3 studies)	The decrease in oxygen saturation was modelled as function of apnoea duration, and the slopes were 0.34, 0.43, and 0.67 for the three studies.
Duration of apnoea with and without desaturation (*n *= 2 studies)	Both studies found that apnoeas with desaturation had longer durations compared to apnoeas without desaturation.
Other metrics (*n* = 7 studies)	Seven studies did not include any of the specific metrics described above. Six of them reported significant relationships, and one reported that no significant dependence of the normalised changes in oxygen saturation on apnoea duration was found.

Five studies^5,21,22,25,52^ presented the mean change or drop in oxygen saturation during apnoea for different apnoea duration subgroups. The average of the mean PMA of participants in the five studies is 32.68 ± 2.48 weeks. In the study by Urlesberger et al.,^25^ none of the participants received respiratory support or supplemental oxygen during the study period. In the study by Finer et al.^21^ supplemental oxygen was administered only to infants whose baseline oxygen saturation fell below 90–92%. For the remaining three studies, information regarding the use of respiratory support or supplemental oxygen was not reported. Meta-analysis pooling the data from these studies showed a negative relationship between apnoea duration and change in oxygen saturation (Fig. 2c, *n* = 5 studies, slope = −0.52, *R*^2^ = 0.95; *r* = −0.98, *p* < 0.0001). Three studies^4,31,48^ used linear regression models to relate apnoea duration and change in oxygen saturation. The regression line slope from the pooled data (−0.51) were within the range of these models (Supplementary Fig. S3, −0.34 for Muttitt et al.^31^ −0.43 for Upton et al.^4^ −0.67 for Upton et al.^48^).

There were 12 other studies^18,30,35–39,45,53–57^ (Table 5, Supplementary Table S1, Supplementary Table S3) which used a variety of oxygen saturation metrics that were not pooled for meta-analyses, such as area under the curve (*n* = 1 study), lowest recorded oxygen saturation (*n* = 1 studies), desaturation incidences (*n* = 2 studies) and duration of apnoeas with and without desaturation (*n* = 2 studies). The data could not be pooled for additional meta-analyses due to limited data availability for each metric. In addition, some studies reported the relationships without numeric values that can be pooled. Most of these studies demonstrated significant correlations between apnoea duration and oxygen saturation, except Curzi-Dascalova et al.^38^ found there were no significant differences between the amplitude of oxygen saturation changes observed following apnoea versus control periods.

### Relationship between apnoea duration and change in cerebral oxygenation

Two studies^5,25^ examined the relationship between apnoea duration and cerebral oxygenation (Supplementary Table S4). Changes in cerebral oxygenation were measured using Near Infra-Red Spectroscopy (NIRS). Urlesberger et al.^25^ and Pichler et al.^5^ both presented the mean change in cerebral haemoglobin difference (cHbD) during apnoea for different apnoea duration groups. Meta-analysis of these two studies demonstrated that cHbD decreased more during apnoeas with longer durations (Fig. 2d, *n* = 2 studies, slope = −0.35, *R*^2^ = 0.83; *r* = −0.92, *p* < 0.0001).

### Relationship between apnoea duration and change in cerebral blood volume

Three studies^5,25,44^ reported the relationship between apnoea duration and cerebral blood volume (Supplementary Table S5). The changes in cerebral blood volume were measured using NIRS. Jenni et al.^44^ reported the correlation coefficients between the amplitude of total haemoglobin concentration (tHb) and duration of apnoea as 0.55 for central apnoea, −0.005 for obstructive apnoea, and 0.7 for mixed apnoea. Urlesberger et al.^25^ found there was no correlation between apnoea and change in concentration changes of total cerebral haemoglobin (cHbtot) for both apnoea duration ≥15 s (*R*^2^ = 0.07 and apnoea duration of 5–14 s (*R*^2^ = 0.01). The tHb and cHbtot are used as surrogate markers of cerebral blood volume. The study by Pichler et al.^5^ had a population comprised of two subgroups: a bradycardia group (bradycardia was defined as a heart rate decrease to below 80 beats per minute, *n* = 20) and a non-bradycardia group (*n* = 19). Episodes of apnoea with associated bradycardia were matched with episodes of apnoea without bradycardia based on the duration and PMA of the neonates. The cerebral blood volume decreased significantly with increased apnoea duration in the bradycardia group, but the relationship was not significant in the non-bradycardia group.

### Factors which modulate the physiological response to apnoea

While most studies showed that longer apnoea durations correspond to greater physiological changes, there is considerable variation, particularly in heart rate changes (Fig. 2). Studies included in this review have investigated whether apnoea type and the use of theophylline can affect the relationship between apnoea duration and changes in heart rate, oxygen saturation, cerebral oxygenation and/or cerebral blood volume. Using meta-analysis, we also explored whether PMA modulates the relationship.

#### Apnoea type

Several studies^31,44,46^ showed that central and obstructive apnoeas could lead to different physiological responses. Muttitt et al.^31^ identified a significant correlation between apnoea duration and heart rate decrease in central apnoeas (computer-diagnosed: *r* = 0.19, *p* < 0.0001; nurse-diagnosed: *r *= 0.20, *p* < 0.0001), but not in obstructive or mixed apnoea groups. Suichies et al.^46^ reported significant positive correlations between the percentage of apnoeas associated with bradycardias and apnoea duration in central apnoeas (*r* = 0.65, *p* < 0.01) and mixed apnoeas (*r* = 56, *p* < 0.05), but the correlation was not significant for obstructive apnoeas. Similarly, Jenni et al.^44^ found that the minimum heart rate during apnoea was negatively correlated with apnoea duration in central apnoeas (*r* = −0.77, *p* < 0.01) and mixed apnoeas (*r* = −0.69, *p* < 0.01) only, but again, no significant correlation was observed in obstructive apnoeas. However, with regard to oxygen saturation, Jenni et al.^44^ observed a significant correlation between apnoea duration and oxygen saturation for all apnoea types (central: *r* = −0.49, *p* < 0.01; obstructive: *r* = −0.49, *p* < 0.05; mixed: *r* = −0.64, *p* < 0.01).

Studies shown in Fig. 2a included four data points associated with central apnoea, one data point associated with obstructive apnoea, and 18 data points involving unspecified apnoea type. Meta-analysis on apnoea type was not performed for these studies due to limited data availability. The meta-analyses of the studies presented in Figs. 2b, c indicated that apnoea type was not a significant modulating factor in the relationship between bradycardia incidence rate and apnoea duration (Supplementary Table S6), but played a significant role in modulating the relationship between changes in oxygen saturation and apnoea duration (Supplementary Table S7).

#### Theophylline

There is limited evidence for theophylline as a modulating factor. Muttitt et al.^31^ found in a sub-group analysis that the correlation between apnoea duration and decrease in heart rate was significant in theophylline-treated group (*r* = 0.16, *p* < 0.0001) but not in the untreated group. Theophylline did not significantly affect the decrease in oxygen saturation in this study. However, the correlation between apnoea duration and decrease in oxygen saturation was the strongest in the theophylline-treated group (*r* = 0.43, *p* < 0.0001). Similarly, Upton et al.^4^ found that theophylline did not reduce the slope of the reduction in oxygen saturation for the duration of the apnoeic attack (treated: *r* = 0.45, *p* < 0.0001; untreated: *r* = 0.24, *p* < 0.0001).

#### Postmenstrual age

While none of the included studies analysed the effect of age on neonates’ responses to apnoea, the meta-analysis of the studies shown in Fig. 2c revealed that PMA significantly modulated the relationship between changes in oxygen saturation and apnoea duration (Supplementary Table S8). A post hoc analysis showed that for neonates with a PMA of 30, 32, 34, and 36 weeks, the predicted apnoea durations associated with a drop in oxygen saturation greater than 10% were 18.9, 20.0, 21.5, and 23.4 s, respectively (i.e. younger neonates have a significant drop in oxygen saturation with shorter apnoeas). In the mixed-effects model analysis comparing percentage change in heart rate with apnoea duration, PMA was not a significant factor (Supplementary Table S9). PMA was not incorporated into the other mixed-effects models due to a lack of information.

### Quality assessment

A summary of the outcomes of the quality assessments can be found in Supplementary Tables S2–S5, and the detailed results from the JBI checklists are provided in Supplementary Tables S10–S13. None of the papers satisfied all the requirements in the checklists. The results of overall risk of bias assessments can be found in Supplementary Table S14. Five studies (12.2%) had a low overall risk of bias, 21 studies (51.2%) had a moderate overall risk of bias, and 15 studies (36.6%) had a high overall risk of bias. Among the 15 studies included in the meta-analyses in Fig. 2 and the combined correlation factors in “Relationship between apnoea duration and change in oxygen saturation”, seven had a high risk of bias, six had a moderate risk, and two had a low risk.

## Discussion

This systematic review and meta-analysis investigated how the duration of apnoea, or pauses in breathing, correlates with changes in heart rate, oxygen saturation, cerebral oxygenation and cerebral blood volume in preterm neonates. Overall, meta-analyses demonstrated that the minimum value and the extent of decrease in heart rate, oxygen saturation, and cerebral oxygenation during apnoea were significantly correlated with the duration of apnoea. This aligns with the outcomes of most studies included in this review. A meta-analysis on cerebral blood volume could not be performed, as the three relevant studies used different metrics. Change in cerebral blood volume was correlated with apnoea duration in the group of neonates with bradycardia in one study, but not in the non-bradycardia group. The other two studies reported surrogate markers for cerebral blood volume, showing inconsistent results—one reported a significant relationship, while the other did not. Due to this variability, further validation is needed, and no definitive conclusion can be drawn from this systematic review regarding the relationship between apnoea duration and changes in cerebral blood volume.

Although there is a strong relationship between physiological instability and apnoea duration, there is nevertheless considerable variation (Fig. 2), with the *R*^2^ values indicating that changes in heart rate exhibited greater variation than changes in oxygen saturation. In the meta-analyses, changes in physiological parameters were assessed as the percentage change during apnoea compared to a baseline period before the apnoea. As neonates generally have a baseline oxygen saturation above 90%, and clinical teams intervene if saturation decreases below 90% for a significant period, the differences in baselines across studies were minimal. In contrast, baseline heart rate can vary much more significantly between individual neonates, which might partly explain this difference in variation between the models. Moreover, differences between studies, such as in measurement techniques or ventilatory support may account for variation.

In addition to variation across studies, there is considerable variation across individual apnoeas, as observed in the included papers, such as Upton et al.^4^ Significant and recurrent physiological fluctuations from apnoea may have clinical implications both acutely and long-term, with studies suggesting associations with increased incidence of ROP^7^ and cognitive deficits later in life.^8,58^ Understanding the factors which modulate the relationship between apnoea duration and physiological instability could allow for the identification of neonates that are at particular risk from large changes in physiology following even short apnoeas, and conversely those neonates who remain physiologically stable. Apnoea type, the use of theophylline and the neonate’s PMA were identified as significant factors modulating the relationship between apnoea duration and changes in heart rate and/or oxygen saturation.

The significant impact of theophylline treatment on modulating the correlation between apnoea duration and heart rate change was reported in only one study.^31^ In that study, a statistically significant correlation was observed in the theophylline-treated group and the combined group, but not in the untreated group. However, these findings should be interpreted with caution. The reported correlations were weak (*r* = 0.093 in the combined group and *r* = 0.16 in the treated group), and no formal statistical comparison between the groups was conducted. A statistically significant result in one subgroup but not in another does not necessarily imply a true difference between the groups. Clinically, it is plausible that theophylline-treated infants primarily experienced apnoea of prematurity, which may involve a more uniform underlying mechanism and thus show clearer associations with bradycardia. In contrast, apnoeas in untreated infants may have stemmed from a wider range of causes such as infection or reflux, leading to more heterogeneous effects on heart rate and obscuring consistent correlations.

Through meta-analyses, we demonstrate that PMA significantly modulates the relationship between apnoea duration and oxygen saturation. A plausible physiological rationale for the observed modulation by PMA is the maturation of the respiratory system and oxygen-handling capacity with increasing postmenstrual age. As PMA increases, functional residual capacity rises,^59^ providing a larger reservoir of oxygen in the lungs at end‑expiration and thereby delaying the onset of desaturation during periods without ventilation. In addition, the transition from predominantly foetal haemoglobin,^60^ which has a high oxygen affinity and is less efficient at tissue oxygen delivery, to adult haemoglobin, which more effectively releases oxygen to tissues, further supports maintenance of tissue oxygenation during apnoeic episodes in older infants. The improved lung compliance, enhanced surfactant production, greater airway stability, and stronger respiratory muscles also contributes to more efficient gas exchange and reduced ventilation–perfusion mismatch. Collectively, these developmental factors likely explain why older preterm infants can tolerate longer apnoeas before exhibiting significant oxygen desaturation. Whilst this finding highlights the potential clinical relevance of age-stratified apnoea alarm thresholds, further research is needed to better understand this relationship in a contemporary cohort of preterm neonates. Our findings provide a foundation for future studies evaluating optimal alarm thresholds in preterm neonates. Such research may ultimately inform clinical guideline development and reduce the burden of false alarms at the bedside, helping staff to prioritise clinically significant events.

Several studies in this review suggest that central apnoeas may be more strongly associated with physiological changes compared to obstructive events, however, these findings should be interpreted cautiously. The evidence base is limited, with relatively few studies systematically distinguishing between apnoea types, and some providing only partial data. As such, future studies are needed to clarify the physiological impact and clinical relevance of obstructive versus central events.

Investigation of other factors modulating the relationship was limited by the number of studies included in this review and the availability of data for each outcome examined. Insufficient data prevented analysis of factors such as sex, pathological conditions, ventilation mode and medications other than theophylline. Moreover, although this review included studies involving various respiratory stimulants, subgroup analysis was only feasible for theophylline due to limited and inconsistent reporting on other methylxanthines such as caffeine. Given that caffeine is now one of the most commonly used respiratory stimulants in neonatal care, future research should aim to investigate the effects of caffeine and other methylxanthines. Inconsistencies in measurement techniques and ventilatory support across the included studies likely contributed to the variability observed in the results. For example, heart rate was measured using a range of modalities, including ECG and plethysmography, which may differ in accuracy and signal resolution. Similarly, definitions and detection thresholds for apnoea, bradycardia, and desaturation varied depending on the monitoring equipment and study protocols. Moreover, differences in ventilatory support, such as whether infants were breathing spontaneously, receiving CPAP, or managed on mechanical ventilation, may have influenced both the physiological response to apnoea and the ability to detect those responses accurately. The length of the observational period for heart rate, oxygen saturation, cerebral oxygenation, and cerebral blood volume following the onset of apnoea may also influence the results. A shorter observational period may fail to capture the full extent of physiological changes, while a longer period risks incorporating unrelated changes. These methodological differences limit the comparability of findings across studies and should be accounted for in future research through standardised measurement protocols and more detailed reporting.

This study was limited by the small sample sizes of most included studies, which may introduce potential bias in the results. Additionally, the publication years of the studies were relatively old, with more than half published over 30 years ago. The methods of measurement and clinical techniques likely differ significantly from those used today. Only five papers showed a low overall risk of bias, while the remaining studies had either moderate or high risks of bias. Common methodological issues included unclear definitions of apnoea and bradycardia, small sample sizes, poor reporting of confounding factors, and limited detail on participant characteristics such as sex or clinical status. For example, less than half of studies (40.9%) reported the sex of the neonates (Supplementary Table S1). To improve the quality and comparability of future research, studies should aim for clearer and standardised definitions of key outcomes, rigorous and transparent reporting of methodology, and prospective designs with adequate sample sizes. Additionally, consistent reporting on modulating factors such as medication use, respiratory support, and baseline physiological status would strengthen the evidence base and enable more refined meta-analyses.

Furthermore, useful information was sometimes presented only in figures, requiring us to extract data points from those figures, which could lead to inaccuracies. The data extraction was performed by a single reviewer, which may introduce the potential for bias. Consistency was prioritised by having one reviewer extract data across all included studies, ensuring uniform application of extraction criteria. Nevertheless, the consistent findings across multiple studies and outcomes in our meta-analyses provide a strong foundation for future work. By identifying PMA as a key modulator of apnoea-related desaturation, this review contributes important insights toward personalising monitoring strategies for preterm neonates and refining clinical approaches to minimise physiological instability.

## Conclusion

This systematic review and meta-analysis provides strong evidence that longer apnoea durations directly correlate with greater deterioration in cardiorespiratory and cerebral parameters in preterm neonates. Most critically, we identified postmenstrual age as a key determinant of physiological vulnerability—with younger infants more likely to experience oxygen desaturation from shorter apnoeas. Current standard monitor settings which usually have the same alarm threshold for all preterm infants are likely inadequate for the most vulnerable babies but conversely may lead to unnecessary alarms in older infants. We recommend further research to evaluate the role of PMA-stratified apnoea alarm thresholds and identify other factors which modulate the relationship between apnoea duration and cardiorespiratory and cerebrovascular changes in infants.

## Supplementary information

Supplementary Material

Supplementary Table 1

PRISMA 2020 Checklist

## Data Availability

All extracted data are provided in Supplementary Table S1.

## Code availability

Code for the meta-analysis can be found at: https://github.com/Yiru-C/apnoea_physiology_meta_analysis.git.
