# Supplementary material for: Apnoea duration and changes in cardiorespiratory and cerebrovascular responses in preterm neonates: a systematic review and meta-analysis
Source: Pediatr Res. 2025 Oct 28;99(6):2179–90. doi: 10.1038/s41390-025-04496-x (PMC13433245; doi:10.1038/s41390-025-04496-x)
Supplement: Supplementary file 1 [file 41390_2025_4496_MOESM1_ESM.pdf]

Supplementary Material: Apnoea duration and changes in cardiorespiratory and cerebrovascular responses in preterm neonates: a systematic review and meta-analysis

Yiru Chen[1], Coen S. Zandvoort[1], Luke Baxter[1], Odunayo A. T. Fatunla[1], Vithushanan Ketheeswaranathan[1], Ravi Poorun[2], Zara Small[3], Fatima Usman[1], Matthew Henry[4], Luc Berthouze[5], Mauricio Villarroel[6], Caroline Hartley[1*]

1. Department of Paediatrics, University of Oxford, Oxford, UK
2. University of Exeter Medical School, University of Exeter, Exeter, UK
3. Medical Sciences Division, University of Oxford, Oxford, UK
4. Bodleian Health Care Libraries, University of Oxford, Oxford, UK
5. Department of Informatics, University of Sussex, Brighton, UK
6. Department of Engineering Science, University of Oxford, Oxford, UK

* Corresponding author:

Dr Caroline Hartley, Department of Paediatrics, University of Oxford, Level 2 Children's Hospital, John Radcliffe, Oxford, OX3 9DU, UK.

Email: caroline.hartley@paediatrics.ox.ac.uk

Supplementary Methods

MEDLINE database via Ovid platform

Medline (Ovid MEDLINE® Epub Ahead of Print, In-Process & Other Non-Indexed Citations, Ovid MEDLINE® Daily and Ovid MEDLINE®) 1946 to present

1 infant/ or infant, newborn/ or infant, low birth weight/ or infant, small for gestational age/ or infant, very low birth weight/ or infant, extremely low birth weight/ or infant, postmature/ or infant, premature/ or infant, extremely premature/ or (infant* or newborn* or new-born* or perinatal* or neonatal* or neo-natal* or postnatal* or post-natal* or baby or babies or neonate* or neo-nate*).ti,ab,kf.

2 apnea/ or breath holding/ or (apno* or apne* or breath hold* or periodic breath* or pause in breath* or cessation of breath* or AOP or respiratory pause* or intermittent breath* or intermittent respiration or inter breath interval* or stop breath*).ti,ab,kf.

3 heart rate/ or bradycardia/ or tachycardia/ or oxygen saturation/ or hypoxia/ or cerebral blood volume/ or brain ischemia/ or hypoxia-ischemia, brain/ or (heart rate* or cardiac rate* or heartbeat* or pulse rate* or bradycardia or tachycardia or oxygen saturation or desat* or oxygen deprivation or lack of oxygen or oxygen starvation or hypoxia or hypoxemia or hypoxeamia or SpO2 or SaO2 or PaO2 or cerebral blood volume or CBV or cerebral blood flow or brain ischemia or cerebral ischeamia or brain blood flow or brain blood supply or brain hemodynamics or brain haemodynamics or brain perfusion or cerebral oxygenation).ti,ab,kf.

4 1 and 2 and 3

5 exp animals/ not humans/

6 4 not 5

Embase database via Ovid platform

Embase 1974 to present

1 infant/ or baby/ or high risk infant/ or hospitalized infant/ or newborn/ or prematurity/ or (infant* or newborn* or new-born* or perinatal* or neonatal* or neo-natal* or postnatal* or post-natal* or baby or babies or neonate* or neo-nate*).ti,ab,kf

2 *apnea/ or *apnea attack/ or *newborn apnea/ or *newborn apnea attack/ or *breath holding/ or (apno* or apne* or "breath hold*" or "periodic breath*" or "pause in breath*" or "cessation of breath*" or AOP or "respiratory pause*" or "intermittent breath*" or "intermittent respiration" or "inter breath interval*" or "stop breath*").ti,ab,kf.

3 heart rate/ or bradycardia/ or tachycardia/ or oxygen saturation/ or hypoxia/ or hypoxemia/ or newborn hypoxia/ or brain blood volume/ or brain ischemia/ or ("heart rate*" or "cardiac rate*" or heartbeat* or "pulse rate*" or bradycardia or tachycardia or "oxygen saturation" or desat* or "oxygen deprivation" or "lack of oxygen" or "oxygen starvation" or hypoxia or hypoxemia or hypoxeamia or SpO2 or SaO2 or PaO2 or "cerebral blood volume" or CBV or "cerebral blood flow" or "brain ischemia" or "cerebral ischeamia" or "brain blood flow" or "brain blood supply" or "brain hemodynamics" or "brain haemodynamics" or "brain perfusion" or "cerebral oxygenation").ti,ab,kf.

4 1 and 2 and 3

5 exp animal/ not human/

6 4 not 5

7 Systematic review.ti,ab. not (trial or study).ti.

8 (review.ab. and review.pt.) not trial.ti.

9 we searched.ab. and (review.ti. or review.pt.)

10 update review.ab.

11 (databases adj4 searched).ab.

12 (conference abstract or conference paper or "conference review" or editorial).pt.

13 7 or 8 or 9 or 10 or 11 or 12

14 6 not 13

PsycINFO database via Ovid platform

PsycINFO 1806 to present

1 premature birth/ or (infant* or newborn* or new-born* or perinatal* or neonatal* or neo-natal* or postnatal* or post-natal* or baby or babies or neonate* or neo-nate*).ti,ab,id.

2 apnea/ or (apno* or apne* or "breath hold*" or "periodic breath*" or "pause in breath*" or "cessation of breath*" or AOP or "respiratory pause*" or "intermittent breath*" or "intermittent respiration" or "inter breath interval*" or "stop breath*").ti,ab,id.

3 heart rate/ or bradycardia/ or tachycardia/ or anoxia/ or cerebral blood flow/ or cerebral ischemia/ or ("heart rate*" or "cardiac rate*" or heartbeat* or "pulse rate*" or bradycardia or tachycardia or "oxygen saturation" or desat* or "oxygen deprivation" or "lack of oxygen" or "oxygen starvation" or hypoxia or hypoxemia or hypoxeamia or SpO2 or SaO2

or PaO2 or "cerebral blood volume" or CBV or "cerebral blood flow" or "brain ischemia" or "cerebral ischeamia" or "brain blood flow" or "brain blood supply" or "brain hemodynamics" or "brain haemodynamics" or "brain perfusion" or "cerebral oxygenation").ti,ab,id.

4 1 and 2 and 3

Cochrane Library platform

#1 [mh ^infant] OR [mh ^"infant, newborn"] OR [mh ^"infant, low birth weight"] OR [mh ^"infant, small for gestational age"] OR [mh ^"infant, very low birth weight"] OR [mh ^"infant, extremely low birth weight"] OR [mh ^"infant, postmature"] OR [mh ^"infant, premature"] OR [mh ^"infant, extremely premature"] OR (infant*:ti,ab,kw OR newborn*:ti,ab,kw OR new-born*:ti,ab,kw OR perinatal*:ti,ab,kw OR neonatal*:ti,ab,kw OR neo-natal*:ti,ab,kw OR postnatal*:ti,ab,kw OR post-natal*:ti,ab,kw OR baby:ti,ab,kw OR babies:ti,ab,kw OR neonate*:ti,ab,kw OR neo-nate*:ti,ab,kw)

#2 [mh ^apnea] OR [mh ^"breath holding"] OR (apno*:ti,ab,kw OR apne*:ti,ab,kw OR ("breath" NEXT hold*):ti,ab,kw OR ("periodic" NEXT breath*):ti,ab,kw OR ("pause in" NEXT breath*):ti,ab,kw OR ("cessation of" NEXT breath*):ti,ab,kw OR AOP:ti,ab,kw OR ("respiratory" NEXT pause*):ti,ab,kw OR ("intermittent" NEXT breath*):ti,ab,kw OR "intermittent respiration":ti,ab,kw OR ("inter breath" NEXT interval*):ti,ab,kw OR ("stop" NEXT breath*):ti,ab,kw)

#3 [mh ^"heart rate"] OR [mh ^bradycardia] OR [mh ^tachycardia] OR [mh ^"oxygen saturation"] OR [mh ^hypoxia] OR [mh ^"cerebral blood volume"] OR [mh ^"brain

ischemia"] OR [mh ^"hypoxia-ischemia, brain"] OR (("heart" NEXT rate*):ti,ab,kw OR ("cardiac" NEXT rate*):ti,ab,kw OR heartbeat*:ti,ab,kw OR ("pulse" NEXT rate*):ti,ab,kw OR bradycardia:ti,ab,kw OR tachycardia:ti,ab,kw OR "oxygen saturation":ti,ab,kw OR desat*:ti,ab,kw OR "oxygen deprivation":ti,ab,kw OR "lack of oxygen":ti,ab,kw OR "oxygen starvation":ti,ab,kw OR hypoxia:ti,ab,kw OR hypoxemia:ti,ab,kw OR hypoxeamia:ti,ab,kw OR SpO2:ti,ab,kw OR SaO2:ti,ab,kw OR PaO2:ti,ab,kw OR "cerebral blood volume":ti,ab,kw OR CBV:ti,ab,kw OR "cerebral blood flow":ti,ab,kw OR "brain ischemia":ti,ab,kw OR "cerebral ischeamia":ti,ab,kw OR "brain blood flow":ti,ab,kw OR "brain blood supply":ti,ab,kw OR "brain hemodynamics":ti,ab,kw OR "brain haemodynamics":ti,ab,kw OR "brain perfusion":ti,ab,kw OR "cerebral oxygenation":ti,ab,kw)

#4 #1 and #2 and #3

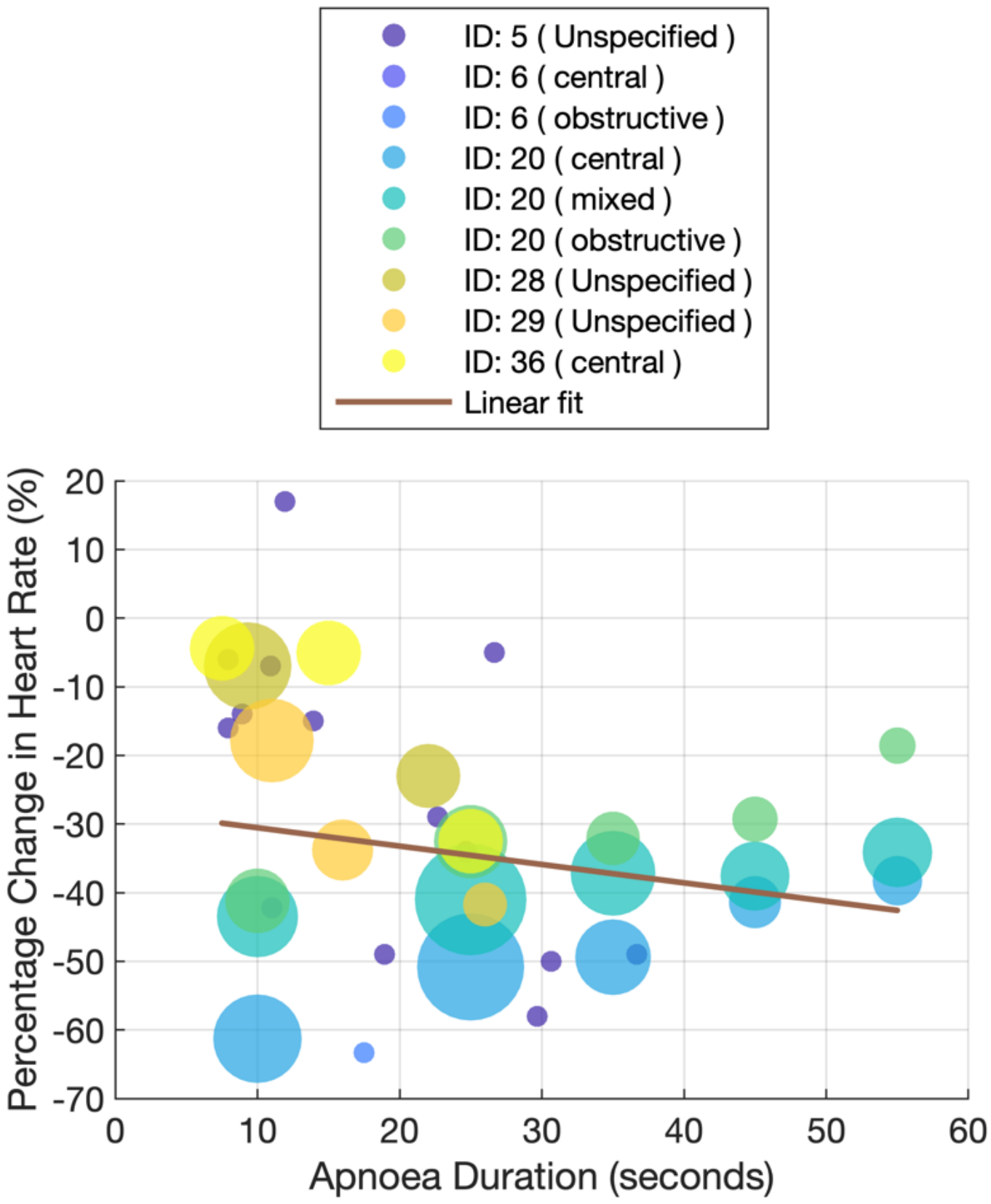

Supplementary Figure 1: The percentage change in heart rate during apnoea on y-axis against apnoea duration on the x-axis. This plot includes the data from Finer et al. (1992). The size of the points indicates their weight in producing the linear best fit, calculated as the square root of the number of apnoea episodes associated with each point. ID: 5 – Fenichel et al. (1980), ID: 6 – Vyas et al. (1981), ID: 28 – Urlesberger et al. (1999), ID: 29 – Carbone et al. (1999), ID: 36 – Beck et al. (2011).

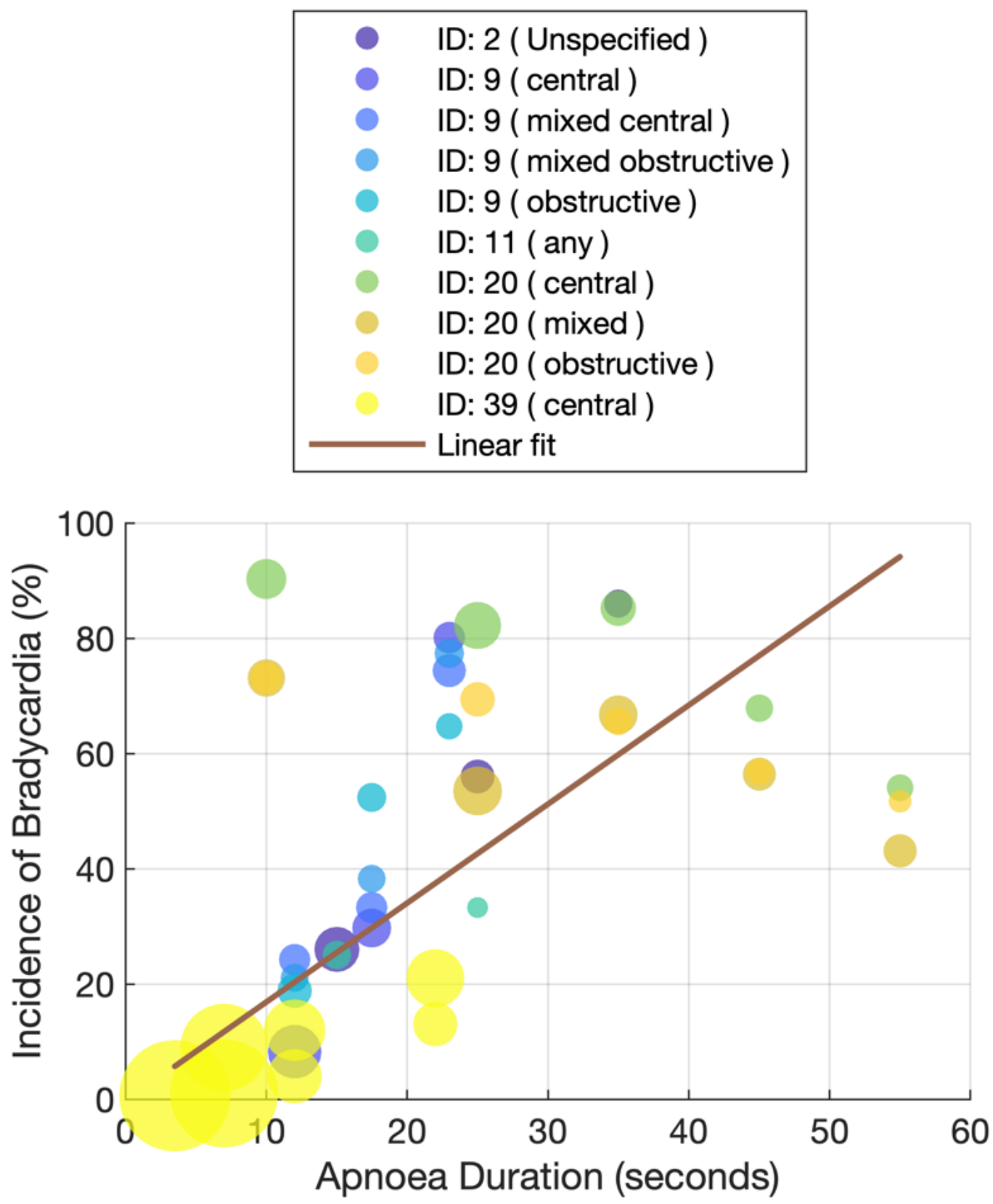

Supplementary Figure 2: The plot displays percentage of apnoea events accompanied by bradycardia on the y-axis against apnoea duration on the x-axis. This plot includes the data from Finer et al. (1992). The size of the points indicates their weight in producing the linear best fit, calculated as the square root of the number of apnoea episodes associated with each point. ID: 2 – Gabriel et al. (1976), ID: 9 - Henderson-Smart et al. (1986), ID: 11 – Mathew (1988), ID: 20 - Finer et al. (1992), ID: 39 – Marshall et al. (2019).

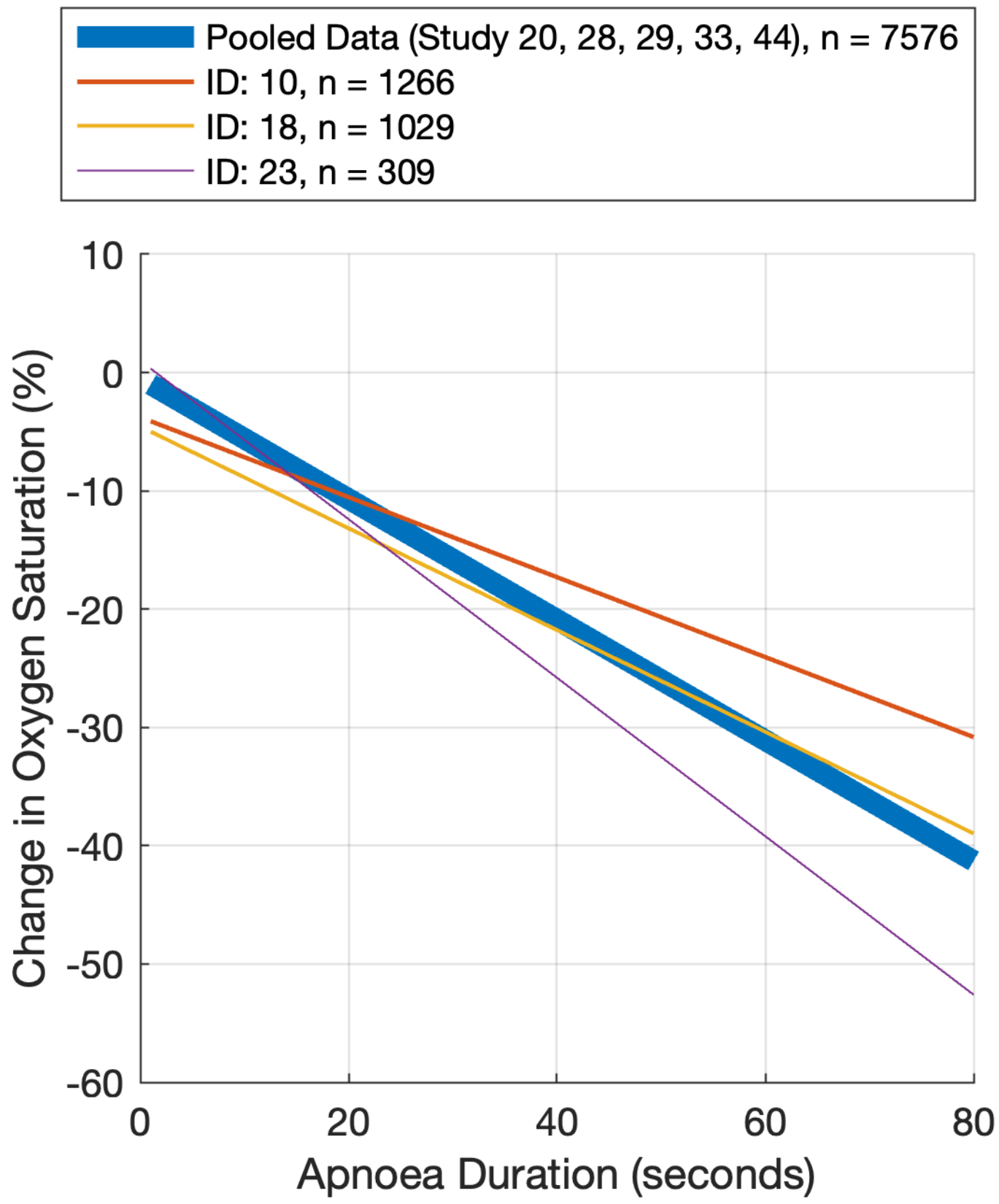

Supplementary Figure 3: This plot displays the change in oxygen saturation on y-axis versus apnoea duration on x-axis. The lines are the best-fit linear regression lines. The three thin lines (red, yellow, purple) represent the regression lines reported by individual studies. The thick blue line represents the regression lines of the data pooled from Gabriel et al. (1976), Henderson-Smart et al. (1986), Mathew (1988), and Marshall et al. (2019). The number n and the thickness of the lines represents the number of apnoea events associated with each line. ID: 10 – Muttitt et al. (1988), ID: 18 – Upton et al. (1991), ID: 23 – Upton et al. (1992).

Supplementary Table 1: Full data set extracted from all the papers included in the review. See the separate excel file. ECG: Electrocardiogram. SD: standard deviation. HR: heart rate. SaO2: oxygen saturation. tHb: total haemoglobin concentration. cHbD: cerebral haemoglobin oxygenation index. cHbtot: concentration changes of total cerebral haemoglobin.

Supplementary Table 2: Summary of included studies reporting the relationship between apnoea duration and change in heart rate. ECG: Electrocardiogram. SD: standard deviation.

Study	Number of infants	Postmenstrual age at study (weeks)	Definition or inclusion criteria of apnoea/pause in breathing	Method of apnoea/pause measurement	Description of apnoea/pause in breathing	Method of outcome measurement	Main results	Quality assessment
Daily et al. 1969	22	Premature	Apnoeic episodes of 20 or more seconds.	Impedance plethysmography (impedance pneumography)	A total of 540 apnoeic episodes were observed in 13 infants. 400 episodes occurred in 8 of 14 infants who were otherwise clinically well. 29 episodes occurred in 4 of the 7 infants recovering from respiratory distress syndrome. Of the 540 episodes, 428 exceeded 30 seconds in duration.	ECG	For apnoea > 45 seconds, heart rate fell steadily; in episodes of shorter duration, there was a tendency for the heart rate to rise in the first 5 seconds and thereafter to fall.	Inclusion criteria was not stated. No confounding factors identified. No statistical analysis performed.
Gabriel et al. 1976 (A)	8	Range: 31 - 35.	Respiratory arrest: pauses > 3 seconds. Apnoeic spells: respiratory arrest of more than 10 seconds duration.	Polysomnography	493 apnoeic spells and 2450 respiratory pauses.	ECG	Pauses < 10 s: no correlation between the duration of the respiratory arrest and the incidence of bradycardia. Pauses >= 10 seconds: the incidence of cardiac slowing as well as the percentage of bradycardia increased with longer duration of apnoeas.	Method of exposure and outcome measurements were unclear. No statistical analysis performed.
Gabriel et al. 1976 (B)	8	Range: 31 - 35.	Breathing pauses are categorised into two groups: pauses lasting 3–10 seconds and apnoeas which exceeded 10 seconds.	Polysomnography	n = 2,450 pauses of 3-10 seconds,<br>n = 493 apnoeas	ECG	Most of the breathing pauses (especially apnoeas) are accompanied by bradycardia. The number of bradycardias increased with increasing apnoea duration.	Method of exposure and outcome measurements were unclear. No statistical analysis performed.
Storrs 1977	9	Range: 27.3 – 34.4.	Apnoea: cessation of spontaneous respiratory effort for > 5 seconds coinciding with cardiovascular changes.	Plethysmography	Total of 47 apnoeas	ECG	The heart rate fell by a mean (±SD) of 43% ± 13%, on average 8.6 seconds ± 6.3 (range 2-33 s) after the onset of apnoea, rising on average 4.8 seconds ± 4.4 (range 2-16 s) after the resumption of breathing. The maximum fall in heart rate is correlated to the apnoea duration.	Method of exposure measurement was unclear. Statistical analysis method was also unclear.

Fenichel et al. 1980	11 newborns, only 6 newborns eligible for this review, the remaining 5 had conceptional age >= 37 weeks.	32; 32; 36; 33; 33; 35.	Not given.	Thermocouple or piezoelectric transducer.	A total of 16 nonconvulsive apnoeic spells were recorded in the eligible patients. Nine of them were between 4-19 seconds, and 7 were >= 20 seconds.	ECG	Alterations in heart rate differed between the two groups (apnoea duration < 20 s and >= 20 s). During apnoea lasting less than 20 seconds, heart rate was found to increase, decrease, or remain unchanged. During apnoea lasting 20 seconds or longer, heart rate usually fell by 40% or more within 8 seconds of the onset of apnoea.	The inclusion criteria and the population details were not clearly stated. The measurement of exposure was unclear. The measurement of outcome was unreliable. Confounding factors were not identified. No statistical analysis performed.
Vyas et al. 1981	7	Mean: 34.1.	Apnoea: cessation of tidal flow for > 5 seconds.	Pneumotachograp h	Total of 172 episodes of apnoea.<br>Number (percentage): obstructive - 86 (50%); central - 48 (27.9%); mixed - 38 (22.1%).<br>Mean apnoea duration (sec): obstructive - 9.93; central - 8.3; mixed - 10.9.	ECG	The lowest heart rate, the mean duration of heart rate below 100 bpm, and the incidences of bradycardia all differed among the three apnoea type groups. Central apnoea tended to have the shortest duration and the smallest changes in heart rate, whereas obstructive and mixed apnoeas were associated with longer durations and greater changes in heart rate.	No statistical analysis performed.
Werthammer et al. 1983	8	Premature.	Apnoea: cessation of respiration for > 15 seconds associated with a decrease in heart rate. Classed as mild when heart rate decreased by 20 beats per minute below the average of the previous two minutes, and as severe when the heart rate fell below 100 beats per minute.	Acoustic monitoring	Total of 26 apnoeas. 17 mild, 9 severe.<br><br>Cessation of breathing (seconds) for the 17 mild episodes: mean 18.2, SD 4.3, range 15-30. Information not given for severe episodes.	ECG	During apnoeic spells that became severe, a mean 27.5 ± 9.7 (SD) seconds elapsed after cessation of respiration before the heart rate fell to less than 100 beats per minute, which is longer than the average duration of mild episodes.	The inclusion criteria and the population details were not clearly stated. Confounding factors were not identified. No statistical analysis performed.

Southall et al. 1983	14 (13 eligible for this review, subject 10 excluded due to infant's age)	Mean: 31.8, range: 28 - 25.	Apnoeic episodes >= 20 seconds and >= 30 seconds.	Impedance respirometer, under-mattress pressure pad, segmented air-filled mattress, pressure capsule system.	A total of 203 apnoeic episodes >= 20 seconds, and 120 episodes >= 30 seconds were identified.<br>Duration of apnoea >= 30 seconds (s): mean: 46, range: 30 - 216.	ECG	Of a total of 203 episodes of apnoea, 145 (71%) were accompanied by bradycardia of 80 bpm.<br>Duration of apnoea is correlated with the duration of accompanying bradycardia.	Confounding factors were not identified. No statistical analysis performed.
Henderson -Smart et al. 1986	28	Range 28 - 44.	All events of 10 seconds or more in duration were noted.	Mercury in rubber strain gauge, thermistor.	There were 1520 apnoeas of over 10 seconds in duration, 1002 with a duration of 10-14 seconds, 311 with duration 15-20 seconds and 207 of more than 20 seconds.	ECG	The major factor influencing the incidence of bradycardia was duration of apnoea. For each apnoea type there was a significant increase in the incidence of bradycardia as duration of apnoea increased ($p<0.001$ in each case). Time to the onset of the fall in oxygen saturation and to the onset of bradycardia became longer with increasing apnoea duration.	The strategies to deal with confounding factors were not stated.
Muttitt et al. 1988	27	Preterm.	The apnoea alarm was set to 15 seconds. This alarm notifies staff of a cessation of respiratory input after 10 seconds by way of an intermittent beep which turns into a continuous alarm after 15 seconds.	Impedance respirometry	A total of 1266 apnoeas were documented in the 27 infants. 28% of the apnoeas were less than 20 seconds in duration, 48% were between 20 and 30 seconds, 21% were between 30 and 50 seconds, and 3% were greater than 50 seconds. The longest lasting recorded apnoea was 104.1 seconds.	ECG	A significant correlation was found between apnoea duration and decrease in heart rate ($r = .093$, $P = .001$).	The inclusion criteria and the population details were not stated. The measurement of outcome was unclear.
Mathew 1988	24	Mean: 35.0, standard deviation: 2.0.	Apnoea: the absence of nasal airflow.<br>Prolonged apnoea: apnoea of 20 seconds or longer duration.<br>Short apnoea: apnoea between 10-19 seconds.	Plethysmography	No episodes of apnoea longer than 10 seconds or bradycardia occurred in 9 infants. 39 episodes of apnoea were observed in the remaining 15 infants.<br>36 episodes were short, 3 prolonged.	ECG	9/36 short apnoeas and 2/3 prolonged apnoeas were associated with bradycardia. Prolonged apnoeas are more likely to be accompanied by bradcardia.	The strategies to deal with confounding factors were not stated. The strategies to address incomplete follow up were not utilised.

Curzi-Dascalova et al. 1989	23 infants recruited: 7 eligible for this review, 16 excluded as had post-conceptional age > 37 weeks).	35-36 week subgroup included in this review	Apnoea: central respiratory pauses > 3 seconds.	Polygraphic recordings	602 pauses in total. None of them lasted more than 12 s.	ECG	Pauses lasting 10 to 12 seconds were relatively rare compared to shorter pauses and did not result in greater cardiac deceleration than shorter pauses.	The inclusion criteria and the population details were not stated. The population details and study settings were unclear. The measurement of exposure was unclear. No statistical analysis performed.
Suichies et al. 1989	18	Preterm	Apnoeic spell: the cessation of breathing for a minimum of 6 seconds.	Thoracic impedance, thermistor	A total of 212 apnoeic spells were analysed. The mean duration of an apnoeic spell was 10.3, 10.2 and 14.3 s for central, obstructive and mixed apnoeic spells, respectively.	ECG	A positive correlation was observed between apnoea duration and the percentage of episodes accompanied by bradycardia. The correlation coefficients varied by apnoea type: central apnoea ($r = 0.65$, $p < 0.01$), obstructive apnoea ($r = 0.51$), and mixed apnoea ($r = 0.56$, $p < 0.05$).	The inclusion criteria and the population details were not stated. The population details and study settings were unclear. The measurement of exposure was unclear. Confounding factors were not identified.
Waggener et al. 1989	14	Premature	Apnoea: cessation of airflow at mask for a minimum of 3 seconds.	Pneumotachograph, magnetometers.	182 apnoeas >=3 s in duration, 38 apnoeas >= 10 in duration.	ECG	The decrease in heart rate during apnoea was modelled as a function of apnoea duration using multiple linear regression and dichotomous logistic regression. The contingency table based on logistic regression showed that 87 out of 99 events with a decrease of <10 bpm and 24 out of 41 events with a decrease of >=10 bpm were successfully predicted, resulting in an average accuracy of 73%.	Confounding factors were not identified. No statistical analysis performed.

Hodgman et al. 1990	66	Range: 32 - 36.	The duration of an apnoeic episode was measured from the end of the inspiration before the pause to the beginning of the next inspiration.	Impedance pneumography.	Approximately half of the infants (53%) had no apnoeic episodes of 15 seconds or longer. 25 (38%) infants had one or more 15- to 19-second apnoea, and in 10 infants (15%), the duration was 20 seconds or longer. Four of these 10 infants (6% of the total population) had two or more episodes of apnoea of this duration.<br>There was a total of 94 individual apnoeic episodes of 15 seconds or longer. Twenty of the 94 episodes lasted 20 seconds or longer, and 5 of these were between 30 and 40 seconds in duration.	ECG	A TEB (transient episodes of bradycardia) was defined as a drop in heart rate below 90 beats per minute. While the majority of TEB did not accompany a long apnoeic episode, all but 5 of the apnoeic episodes of 15 seconds or longer were accompanied by a TEB.	The measurement of exposure was unclear. Confounding factors were not identified. Methods of statistical analysis were unclear.
Mathew et al. 1991	10	Preterm.	Apnoeic episodes: absence of airflow for 5 seconds or greater.<br>Apnoeas lasting >= 15 seconds were termed long apnoeas, whereas those lasting < 15 seconds were termed short apnoeas.	Plethysmography, nasal Screen Flowmeter	A total of 352 apnoeic events. Out of the 202 episodes preceded by arousal, most of the apnoeas were short: 181 were less than 15 s, and only 21 were > 15 s.	ECG	Hypoxia or bradycardia developed during 24% of long apnoea (>= 15 s) and only 7% of short apnoea (< 15 s).	The inclusion criteria and the population details were not stated. Confounding factors were not identified. No statistical analysis performed.
Upton et al. 1991	27	Median: 32, range: 26 - 36.	Apnoea: cessation of breathing movement for at least 10 seconds, or a shorter period if associated with bradycardia of 90 beats per minute or less.	Thoracic impedance, plethysmography.	A total of 1029 episodes were analysed.<br>Duration of apnoea (s): median 10 (range 0-80) for infant receiving theophylline (45 studies, 535 episodes); median 11 (range 0-31) for infants not receiving theophylline (44 studies, 494 episodes).	ECG	Correlation between minimum heart rate during episodes of bradycardia and apnoea is extremely poor. Minimal heart rate=74.22-0.14*duration of apnoea (r=0.11, p<0.0001).	The measurement of exposure was unclear. The strategies to deal with confounding factors were not stated.

Spear et al. 1992	26	All patients - mean: 34, SD: 2.<br><br>Patients with prolonged apnoeas - mean 33, SD: 1.<br><br>Patients with no prolonged apnoeas - mean: 35, SD: 2.<br><br>p = 0.001.	Prolonged apnoea: > 20 seconds, short apnoea: 6 - 20 seconds.	Thoracic impedance, nasal air flow (by thermistor).	Of the 78 traces, 20 had prolonged apnoea and 58 had no prolonged apnoea.<br><br>Traces had a mean of 16.1 episodes of central apnoea, 3.04 episodes of obstructive apnoea, and 6.1% of periodic breathing.	ECG	The number of bradycardias > 5 seconds were greater in the traces with prolonged apnoea comparing to short apnoea.	The two groups were not similar in study weight, post-conceptional age and postnatal age. No statistical analysis performed.
Finer et al. 1992	47	Premature	Apnoea was included in this study if it lasted at least 15 seconds and was associated with bradycardia, hypoxaemia, or both.	Impedance respirometry	2082 episodes of apnoeas in total.	ECG	As apnoea duration increased, the percentage fall in heart rate decreased, this trend being significantly greater for central and obstructive apnoea than for mixed events. As the duration of central apnoea became longer, untreated infants had a significantly lesser fall in heart rate; apnoea between 40 and 50 seconds in duration was associated with a mean fall in heart rate of 38%, compared with 51% during apnoea lasting 20 to 30 seconds (p = <0.0001).	The inclusion criteria and the population details were not stated. The details of the study subjects and the setting were unclear (study period was not stated). The measurement of exposure was unclear. Confounding factors were not identified.

Upton et al. 1992 (A)	27 (24 have available flow recordings).	Median: 32, range: 26 - 36.	Apnoeic episodes of at least 5 seconds duration, as detected on the flow trace, were analysed for comparison.	Thoracic impedance, plethysmography.	From the initial polygraphic recording: 86% of the bradycardic episodes were associated with apnoea.<br><br>During airway flow measurements: apnoea was present on the flow trace in all 41 episodes in which bradycardia occurred. Median apnoea duration was 14 seconds (range 2-51).	ECG	The duration of bradycardia, expressed by the number of bradycardic beats, was positively correlated with apnoea duration in seconds. There was also a significant positive correlation between apnoea duration and time of onset of bradycardia.	The measurements of exposure and outcome were unclear.
Poets et al. 1993	80	Mean: 36.3, SD: 2.3.	Apnoeic pauses of 4 seconds or longer associated with bradycardia. An association was considered present if commenced within ±15 seconds of the onset of the bradycardia.	Volume expansion capsule, plethysmography.	143 (83%) of the 172 bradycardias associated with interpretable airflow and breathing movement signals were accompanied by an apnoeic pause.	ECG	The apnoeic pauses associated with bradycardia had a median duration of 10 seconds, with range between 4 and 38 seconds.	The inclusion criteria and the population details were not stated. The details of the study subjects and the setting were unclear (study period was not stated). The measurements of exposure were unclear. Confounding factors were not identified. No statistical analysis performed.
Jenni et al.1996	17	Preterm	Apnoea: cessation of respiratory effort for 10 seconds or more.	Thoracic impedance, thermistor.	A total of 130 apnoeic episodes were recorded in 14 infants. The median duration of those episodes was 20 s, the 5th and 95th percentiles were 12 and 54, respectively. Each infant had between 4 and 20 episodes of apnoea (mean 9.3) per study period).	ECG	The duration of apnoea was correlated with the lowest heart rate: central apnoea: $r = -0.772$ ($p < 0.01$), obstructive apnoea: $r = 0.104$, and mixed apnoea: $r = -0.695$ ($p < 0.01$).	The inclusion criteria and the population details were not stated. The measurements of exposure were unclear. Confounding factors were not identified.

Urlesberger et al. 1999	58	Preterm	Apnoeas were divided into 2 groups according to their duration: Group 1 included apnoeas with a duration between 5-14 seconds, and Group 2 included apnoeas >= 15 seconds. If there were changes in oxygen saturation to a degree of >= 3%, and/or in heart rate to a degree of >= 5% of baseline values in either direction, the episode was placed in Subgroup b, otherwise the episode was placed in Subgroup a.	2 peizo respiratory effort sensors.	A total number of 1345 apnoeas in Group 1 and 74 apnoeas in Group 2 were recorded. In Group 1, 587 apnoeas (44%) fulfilled criteria for further analysis, 382 of them were in Subgroup 1a, and 205 were in 1b. In Group 2, 60 apnoeas (81%) fulfilled criteria for further analysis, all of them were placed into Subgroup 2b.	ECG	Group 2b has a longer mean duration of apnoeas and a greater decrease in heart rate comparing to Group 1b.	The exposures were not measured similarly to assign people to both exposed and unexposed groups. The measurements of exposure were unclear. Confounding factors were not identified. Participants were not free of the outcome at the start of the study. Lack of information on follow-up time.
Carbone et al. 1999	90 infants recruited, multichannel recording data were complete in 32 infants.	Mean: 36.9.	Episodes of apnoea of >= 10 seconds in duration.	Thermistor.	236 episodes of apnoea analysed. The long apnoea group consisted of 14 apnoeic events of >=20 seconds in duration, the medium apnoea group consisted of 50 apnoeic events of 15 to 19 seconds in duration, and the short apnoea group consisted of 172 apnoeic events of 10 to 14 seconds in duration.	Monitor	Longer apnoeas were significantly correlated with a lower heart rate nadir, a longer duration of heart rate below two-thirds of the baseline, a greater drop in heart rate, and a faster rate of heart rate decline.	The inclusion criteria and the population details were not stated. The details of the study subjects and the setting were unclear (study period was not stated). The measurements of exposure were unclear. Confounding factors were not identified. Methods of statistical analysis were unclear.

Curzi-Dascalova et al. 2000	5	Mean: 34, range: 33.6 - 34.7.	Apnoea: a 20% or greater decrease in the amplitude of abdominal respiratory movements (detected 2 cm above the umbilicus) or/and airflow.	Thermistors.	492 apnoeas longer than 5 s were detected, of those only 27 were longer than 10 s.	ECG	Multivariate linear analysis showed that the normalised percentage change in HR was dependent on apnoea duration ($p < 0.0001$); regression analysis models explained 24.7% of the changes found, and coefficient of correlation was $r = 0.50$.	The details of the study subjects and the setting were unclear (study period was not stated). The measurements of exposure were unclear.
Pichler et al. 2003	Bradycardia group: n = 20; Non-Bradycardia group: n = 19.	Bradycardia group - mean: 34.2, SD: 2.8.<br><br>Non-Bradycardia group - mean: 35.0, SD: 2.4.<br><br>p value > 0.05	Episodes of apnoea lasting longer than 15 s with bradycardia (bradycardia group) were analysed and compared to episodes of apnoea lasting longer than 15 s without bradycardia (non-bradycardia group).	Piezo-respiratory effort sensors	Twenty-six episodes of apnoea with bradycardia and 26 without bradycardia were observed. The mean duration of apnoea episodes was 25.9 ± 6.0 s in the bradycardia group and 25.0 ± 8.6 s in the non-bradycardia group ($p > 0.05$).	ECG	Longer apnoea durations are associated with greater drops in heart rate in both the bradycardia and non-bradycardia groups.	Unclear whether the two groups were similar and recruited from the same population. Confounding factors were not identified.
Nock et al. 2004	17	Mean: 35, SD: 1.	Number of apnoeas >=15 seconds and >=20 seconds were recorded. Those lasting >=20 seconds are a subset of the episodes >=15 seconds in length.	Plethysmography.	The subjects had 0 to 46 apnoeic episodes >=15 seconds and 0 to 14 episodes >=20 seconds.	ECG	Heart rate was 119 ± 17 (94-163) bpm during apnoeas >=15 seconds and 110 ± 11 (91-126) bpm during apnoeas >=20 seconds.	The details of the study subjects and the setting were unclear (study period was not stated). The measurements of exposure were unclear. Confounding factors were not identified.

Beck et al. 2011	10	Mean: 32.1, range: 29.6 - 36.4.	Central apnoeas: periods where the electrical activity of the diaphragm was a flat tracing for more than 5 seconds.	Oesophageal electromyography .	188 central apnoeas identified. Apnoeas were classified by their duration (5 - 10 s, 10 - 15 s, and > 15 s).	Not given	Longer apnoea durations were associated with greater drops in heart rate during the events.	The details of the study subjects and the setting were unclear (study period was not stated). The strategies to deal with confounding factors were not stated.
Mohr et al. 2015	335	Preterm	ABD: Apnoea events accompanied by bradycardia (heart rate <100 bpm) and oxygen desaturation (oxygen saturation <80%). ABD events with apnoea lasting more than 10 seconds were recorded and categorised by duration.<br>Very long apnoea (VLA) was defined as breathing cessation >60 seconds with bradycardia and oxygen desaturation.	Chest impedance-derived respiratory rate by central network server.	86 VLA occurred in 29 infants. The number of events per infant ranged from 1 to 19.<br>In all infants there were 100 events of a duration 10-20 seconds, 100 events of 30-40 seconds, 46 events of 60-80 seconds, and 40 events of 80 seconds.	ECG	The areas under the HR curves increase approximately linearly with the duration of the apnoea. The sample correlation coefficient is 0.81 for heart rate ($p < 0.001$).	The inclusion criteria and the population details were not stated. Confounding factors were not identified.
Marshall et al. 2019	30	Median: 30, inter-quartile range: 28-33.	Individual respiratory pauses between 5 and 29 seconds (inclusive) were identified. Events with an FiO2 (fraction of inspired oxygen) adjustment (defined as a change in FiO2 of at least 0.01) occurring during the event window were excluded.	Abdominal capsule.	In total, 17677 isolated and 9623 clustered respiratory pause events were identified from a total of 55767 respiratory pauses greater than or equal to 5 seconds. After exclusion of 572 isolated and 443 clustered events because of FiO2 adjustments within the event window, there were 17105 isolated and 9180 clustered events available for analysis.	Monitor	The mean trajectories of heart rate over the event window demonstrate more prominent instability after longer duration pauses, both for pauses that were isolated and clustered. The proportion of events followed by physiological instability increased with longer pause duration, and was higher for clustered events for all pause durations.	The exposure and outcomes were measured in valid and reliable ways. Confounding factor (clustering of apnoea) and the strategy to deal with the factor were stated.

Falsaperla et al. 2019	1	29.6	Apnoea: cessation of airflow lasting more than 20 seconds, or cessation of airflow associated with bradycardia (20% below the baseline heart rate), or cessation of airflow for less than 20 seconds with oxygen desaturation less than 80%.	Polygraphic recordings	Number of apnoeic episodes: 8 per day. Duration of episodes: 20-28 secs.	Polygraphic recordings	After about 10 s of central apnoea an abrupt decrease of the child's heart rate (more than 50% variation, from 160 bpm to 65 bpm) was recorded. The drop in the heart rate was followed by a new onset of thoracic efforts, associated with mixed central apnoea at the final stage, which lasted about 10 s, and then the episode resolved with a clear end-point.	The diagnostic tests and the results were not clearly described. The intervention and the post-intervention clinical condition were unclear. The adverse events were not identified.
Seppa-Moilanen et al. 2021	21	Median: 35.7, inter-quartile range: 35.0 - 36.3	Apnoea: central respiratory pauses longer than two breathing cycles and lasting 4 seconds or more. All obstructive breaths were noted regardless of the duration of apnoea.<br><br>AOP: an apnoea with a heart rate decrease to <100 beats/min, or a drop in oxygen saturation to under 80%, or it lasted longer than 20 s.	Nasal airflow by a pressure sensor, abdominal band.	Total of 3993 apnoeas in the recordings.	Monitor	For mixed apnoea, events accompanied by arousal were associated with longer durations and higher heart rates. In AOP, events with arousal had longer durations and lower heart rates. For mixed apnoea AOP, events with arousal exhibited longer durations but similar heart rates.	The details of the study subjects and the setting were unclear (study period was not stated).

Supplementary Table 3: Summary of included studies reporting the relationship between apnoea duration and change in oxygen saturation. SD: standard deviation.

Study	Sample size	Postmenstrual age at study (weeks)	Definition or inclusion criteria of apnoea/pause in breathing	Method of apnoea/pause measurement	Description of apnoea/pause in breathing	Method of outcome measurement	Main results	Quality assessment
Muttitt et al. 1988	27	Preterm.	See Supplementary Table 2.	See Supplementary Table 2.	See Supplementary Table 2.	Pulse oximetry	A significant correlation was found between apnoea duration and decrease in oxygen saturation in the total population of infants with apnoea (r = .388, P < .001). Regression equation: percentage decrease in oxygen saturation = 3.778 + 0.338 * duration of apnoea.	See Supplementary Table 2.
Poets et al. 1991	31 (16 cases, 15 control)	Cases - mean: 40, range: 36 - 47.<br><br>Control - mean: 40, range: 35 - 45.	Apnoeic pauses: a cessation in breathing movements for at least 4 seconds.<br><br>Periodic breathing: a sequence of at least three apnoeic pauses, each separated by not more than 20 breaths.	Graseby pressure capsule, respitrace, thermistor.	Apnoea duration with episodes (s):<br><br>Cases:<br>periodic - median: 7.8, range: 6.2 - 19.2.<br>non-periodic - median: 11.9, range: 7.3 - 21.0.<br><br>Controls:<br>periodic - median: 5.4, range: 4.0 - 5.6.<br>non-periodic - median: 6.0, range: 4.0 - 8.5.	Pulse oximetry	The mean duration of apnoeic pauses associated with a hypoxaemic episode was significantly shorter during periodic breathing.	Unclear whether the two groups were comparable. Unclear whether the same criteria used for identification of cases and controls. Confounding factors were not identified.
Mathew et al. 1991	10	Preterm.	See Supplementary Table 2.	See Supplementary Table 2.	See Supplementary Table 2.	Pulse oximetry	Hypoxia or bradycardia developed during 24% of long apnoea (>= 15 s) and only 7% of short apnoea (< 15 s).	See Supplementary Table 2.

Upton et al. 1991	27	Median: 32, range: 26 - 36.	See Supplementary Table 2.	See Supplementary Table 2.	See Supplementary Table 2.	Pulse oximetry	There was a positive correlation between the reduction in oxygen saturation and the duration of apnoea. Reduction in oxygen saturation = 4.57+0.43*duration of apnoea ($r=0.41$, $p<0.0001$).	See Supplementary Table 2.
Spear et al. 1992	26	See Supplementary Table 2.	See Supplementary Table 2.	See Supplementary Table 2.	See Supplementary Table 2.	Pulse oximetry	Total number of desaturations < 90%, desaturations < 80% and < 90% in association with apnoea and/or bradycardia, longest duration of desaturation < 80%, and total duration of desaturation < 90% during sleep were all greater in the tracings with prolonged apnoea.	See Supplementary Table 2.
Finer et al. 1992	47	See Supplementary Table 2.	See Supplementary Table 2.	See Supplementary Table 2.	See Supplementary Table 2.	Pulse oximetry	As apnoea duration increased, the fall in oxygen saturation also increased ($r = 0.26$; $p = 0.0001$); this relationship was significant for all apnoea types and did not differ significantly between apnoea types or between treatment groups. There was a significant association between the pre-apnoea mean oxygen saturation and apnoea duration ($r =-0.14$; $p <0.0001$).	See Supplementary Table 2.

Abdulhamid et al. 1992	91 enrolled, 43 patients were <37 weeks gestation, and 25 of these were < 37 weeks postconceptional age at the time of evaluation.	Mean: 40.5, range: 34 - 88.<br><br>25 patients < 37.	central apnoea >= 20 seconds;<br>central apnoea < 20 seconds associated with bradycardia < 80 bpm for > 5 seconds in preterm infants .	Thoracic impedance, nasal / oral airflow.	A total of 184 episodes were recorded.	Pulse oximetry	The duration of obstructive apnoea ranged from 11 to 27 seconds, with 118 episodes (94%) associated with desaturation levels in the 71–84% range. Mixed apnoea episodes lasted between 10 and 45 seconds, with 25 episodes (47%) linked to desaturation levels in the 60–84% range.	The inclusion criteria and the population details were not stated. The details of the study subjects and the setting were unclear (study period was not stated). Confounding factors were not identified. Methods of statistical analysis were unclear.
Upton et al. 1992 (B)	24	Median: 32, range: 26 - 36.	Apnoea: cessation of airflow for 5 seconds or longer.	Pneumotachograph, plethysmography, thoracic impedance.	309 apnoeas. Episodes occurred in 60 of the 83 studies, with a median two apnoeas per study (range 0-19).<br><br>Mean apnoea duration recorded was 8.3 seconds (range 5-51 seconds).	Pulse oximetry	Positive correlation between oxygen saturation drops during apnoea and apnoea duration (oxygen saturation drop = (apnoea duration * 0.67) - 1.0, r = 0.75, p < 0.0001).	Unclear whether the participants were free of outcomes at the start of the study.
Poets et al. 1993	80	Mean: 36.3, SD: 2.3.	See Supplementary Table 2.	See Supplementary Table 2.	See Supplementary Table 2.	Pulse oximetry	The interval between onset of apnoeic pause and onset of desaturation has a median of 5.2 seconds, ranged between 0 and 15 seconds.	See Supplementary Table 2.
Poets et al. 1995	96	Median: 34, range: 29 - 37.	Apnoeic pause of 20 seconds or longer associated with desaturations were recorded.<br>An association was considered present if the apnoeic pause began within 12 seconds of the desaturation.	Volume expansion capsule.	23 desaturations in 7 infants were associated with prolonged apnoeic pauses (>= 20 seconds). The duration of these pauses ranged from 21 to 120 seconds (median, 45 seconds).	Pulse oximetry	There was no significant correlation between the duration of the apnoeic pause and that of the desaturation (r = 0.3; P > .05, Spearman's rank correlation test).	The details of the study subjects and the setting were unclear (study period was not stated). The measurements of exposure were unclear. The strategies to deal with confounding factors were not stated.

Jenni et al. 1996	17	Preterm	See Supplementary Table 2.	See Supplementary Table 2.	See Supplementary Table 2.	Pulse oximetry	The duration of apnoea was correlated with the lowest oxygen saturation as follows: central apnoea: $r = -0.486$ ($p < 0.01$), obstructive apnoea: $r = -0.491$($p < 0.005$), and mixed apnoea: $r = -0.637$ ($p < 0.01$).	See Supplementary Table 2.
Adams et al. 1997	30	Mean: 32.8, SD: 2.3.	The duration of all respiratory pauses that led to a fall in oxygen saturation of < 80% for > 4 seconds.	Plethysmography, thermistor.	156 pauses were recorded (40 apnoeas > 15s, 22 pauses with lower EELV (end-expiratory lung volume), 94 pauses with unchanged EELV)	Pulse oximetry	There was no significant correlation between the length of the respiratory pauses and the rate of desaturations ($r = -0.198$)	The inclusion criteria and the population details were not stated. Confounding factors were not identified. The method of statistical analysis was unclear.
Urlesberger et al. 1999	58	Preterm	See Supplementary Table 2.	See Supplementary Table 2.	See Supplementary Table 2.	Pulse oximetry	Group 2b has a longer mean duration of apnoeas and a greater decrease in oxygen saturation comparing to Group 1b.	See Supplementary Table 2.
Carbone et al. 1999	90 infants recruited, data were complete in 32 infants.	Mean: 36.9.	See Supplementary Table 2.	See Supplementary Table 2.	See Supplementary Table 2.	Pulse oximetry	Longer apnoeas were significantly correlated with a lower saturation nadir, a longer duration of saturation < 85%, a greater drop in saturation and a faster rate of saturation decline.	See Supplementary Table 2.

Curzi-Dascalova et al. 2000	5	Mean: 34, range: 33.6 - 34.7.	See Supplementary Table 2.	See Supplementary Table 2.	See Supplementary Table 2.	Pulse oximetry	There were no significant differences between the amplitude of SaO2 changes observed following apnoea versus control periods. Linear mixed model analysis failed to demonstrate any significant dependence of the normalised changes in oxygen saturation on apnoea duration.	See Supplementary Table 2.
Pichler et al. 2003	See Supplementary Table 2.	See Supplementary Table 2.	See Supplementary Table 2.	See Supplementary Table 2.	See Supplementary Table 2.	Pulse oximetry	Longer apnoea durations are associated with greater drops in arterial oxygen saturation in both the bradycardia and non-bradycardia groups.	See Supplementary Table 2.
Nock et al. 2004	17	Mean: 35, SD: 1.	See Supplementary Table 2.	See Supplementary Table 2.	See Supplementary Table 2.	Pulse oximetry	Oxygen saturation during spontaneous apnoeas >= 15 seconds fell to 86 ± 9%, mean ± SD (range, 62%-99%). During apnoeic episodes >= 20 seconds, saturation fell to 81 ± 11% (58%-92%).	See Supplementary Table 2.
Cardot et al. 2007	36	Premature	respiratory pause lasting > 3 seconds.	Thoracic impedance, pneumotachograph.	Mean apnoea duration was 5.5 ± 1.3 seconds.	Pulse oximetry	The duration of apnoeas with desaturation (6.3 ± 2.0 seconds) was longer compared to the duration of apnoeas without desaturation (5.2 ± 1.0 seconds).	The inclusion criteria and the population details were not stated. The details of the study subjects and the setting were unclear (study period was not stated). The measurements of exposure were unclear. The strategies to deal with confounding factors were not stated.

Tourneux et al. 2008	29	Late preterm.	Central apnoeic events were defined as respiratory pauses lasting more than 3 seconds.	Plethysmography.	About 1900 apnoeic events were analysed. All lasted less than 20 s.	Pulse oximetry	Apnoeas with desaturation had longer durations compared to apnoeas without desaturation.	The inclusion criteria and the population details were not stated. The details of the study subjects and the setting were unclear (study period was not stated). The strategies to deal with confounding factors were not stated.
Elder et al. 2011	Cases (n = 16). Controls (n = 18).	Cases - median: 38, range: 35 - 45.<br><br>Controls - median: 37, range: 35 - 44.	Apnoea: cessation of nasal/oral airflow for >= 5 seconds or a pause of < 5 seconds if there was a drop in oxygen saturation to < 95% or an associated 3% desaturation.	Thermistor.	Mean apnoea length (s): cases 6.9; controls 6.4. Longest apnoea (s): cases 17.4; controls 12.3. (Log-transformed, values expressed as geometric mean.)	Pulse oximetry	For all infants, there was a significant correlation between the lowest recorded oxygen saturation in active sleep for each infant and the longest apnoea duration recorded for each infant (r = -0.530, p = 0.002). This remained significant when group status was controlled for (r = -0.436, p = 0.01).	The groups were not comparable other than the presence of disease in cases or the absence of disease in controls. The measurements of exposure were unclear. Confounding factors were not identified. No statistical analysis performed.

Mohr et al. 2015	335	Preterm	See Supplementary Table 2.	See Supplementary Table 2.	See Supplementary Table 2.	Pulse oximeter	The area under the oxygen saturation curve increased approximately linearly with the duration of the apnoea (correlation coefficient: 0.88, $p < 0.001$).	See Supplementary Table 2.
Fathabadi et al. 2017	20	Preterm	Apnoea: a pause in respiration of 4 seconds or greater.	Respiratory motion.	Apnoea occurred concomitant with the hypoxic event in 555 (45 %) cases, including 495 (40 %) in which apnoea preceded the event and 165 events in which it occurred concurrently/subsequently (105 hypoxic events had both preceding and concurrent/subsequent apnoea).	Pulse oximetry	Longer apnoea durations were associated with longer hypoxia durations and a smaller oxygen saturation slope.	Confounding factors were not identified. Unclear whether the participants were free of the outcome at the start of the study. The measurements of outcomes were unclear.
Marshall et al. 2019	30	Median: 30, inter-quartile range: 28-33.	See Supplementary Table 2.	See Supplementary Table 2.	See Supplementary Table 2.	Pulse oximeter	In pooled data, the mean trajectories of oxygen saturation over the event window demonstrate more prominent instability after longer duration pauses, both for pauses that were isolated and clustered. The proportion of events followed by physiological instability increased with	See Supplementary Table 2.

							longer pause duration, and was higher for clustered events for all pause durations.	
Seppa-Moilanen et al. 2021	21	Median: 35.7, inter-quartile range: 35.0 - 36.3	See Supplementary Table 2.	See Supplementary Table 2.	See Supplementary Table 2.	Pulse oxymeter	For central apnoea, events accompanied by arousal were associated with similar durations and higher oxygen saturation. For mixed apnoea, AOP and mixed apnoea AOP, events accompanied by arousal were associated with longer durations and higher oxygen saturation.	See Supplementary Table 2.

Varisco et al. 2022	26	Mean: 29.2.	CASEs: Count of central apnoea-suspected events. CASEs were then divided and grouped according to the length of their cessation of breathing L. The following lengths L were considered: 10s <= L < 20s, 20s <= L < 30s, 30s <= L < 40s, 40s <= L < 50s, 50s <= L < 60s , 60s <= L < 80s , L >= 80s.	Chest impedance.	A total number of 4756 CASEs were recorded. Number of CASEs for 10s <= L < 20s: 3140; 20s <= L < 30s: 655; 30s <= L < 40s: 404; 40s <= L < 50s: 202; 50s <= L < 60s: 106; 60s <= L < 80s: 127; L >= 80s: 122.	Pulse oxymeter	Longer apnoea durations were associated with greater decrease in oxygen saturation. The decrease was 5%, 12%, 17%, 22%, 27%, 32% and 43% for apnoea lengths of 10, 20, 30, 40, 50, 60 and 80 s, respectively.	The details of the study subjects and the setting were unclear (study period was not stated). The strategies to deal with confounding factors were not stated. No statistical analysis performed.

Supplementary Table 4: Summary of included studies reporting the relationship between apnoea duration and change in cerebral oxygenation. cHbD: cerebral haemoglobin oxygenation index.

Study	Sample size	Postmenstrual age at study (weeks)	Definition or inclusion criteria of apnoea/pause in breathing	Method of apnoea/pause measurement	Description of apnoea/pause in breathing	Method of outcome measurement	Main results	Quality assessment
Urlesberger et al. 1999	58	Preterm	See Supplementary Table 2.	2 piezo respiratory effort sensors.	See Supplementary Table 2.	Near-Infrared Spectroscopy	There was poor correlation between apnoea length >= 15 sec and delta cHbD (R^2 = 0.27). There was no correlation between an apnoea length of 5-14 sec and delta cHbD (R^2 = 0.06).	See Supplementary Table 2
Pichler et al. 2003	See Supplementary Table 2.	See Supplementary Table 2.	See Supplementary Table 2.	See Supplementary Table 2.	See Supplementary Table 2.	Near-Infrared Spectroscopy	Longer apnoea durations are associated with greater drops in cHbD in both the bradycardia and non-bradycardia groups.	See Supplementary Table 2.

Supplementary Table 5: Summary of included studies reporting the relationship between apnoea duration and change in cerebral blood volume. tHb: total haemoglobin concentration. cHbtot: concentration changes of total cerebral haemoglobin.

Study	Sample size	Postmenstrual age at study (weeks)	Definition or inclusion criteria of apnoea/pause in breathing	Method of apnoea/pause measurement	Description of apnoea/pause in breathing	Method of outcome measurement	Main results	Quality assessment
Jenni et al. 1996	17	Preterm	See Supplementary Table 2.	See Supplementary Table 2.	See Supplementary Table 2.	Near-Infrared Spectroscopy	The duration of apnoea was correlated with the amplitude of tHb as follows: central apnoea: $r = 0.549$ ($p < 0.01$), obstructive apnoea: $r = -0.005$, and mixed apnoea: $r = 0.7$.	See Supplementary Table 2.
Urlesberger et al. 1999	58	Preterm	See Supplementary Table 2.	2 piezo respiratory effort sensors.	See Supplementary Table 2.	Near-Infrared Spectroscopy	There was no correlation between apnoea length >= 15 sec and delta cHbtot ($R2 = 0.07$). There was no correlation between an apnoea length of 5-14 sec and delta cHbtot ($R2 = 0.01$).	See Supplementary Table 2
Pichler et al. 2003	See Supplementary Table 2.	See Supplementary Table 2.	See Supplementary Table 2.	See Supplementary Table 2.	See Supplementary Table 2.	Near-Infrared Spectroscopy	Longer apnoea durations are associated with greater drops in cerebral blood volume (CBV) in the bradycardia group, but not in the non-bradycardia group.	See Supplementary Table 2.

Supplementary Table 6: Results of the statistical analysis investigating if apnoea type modulates the relationship between bradycardia incidence and apnoea duration. Fixed effects coefficients from the linear mixed-effects model with the formula: "bradycardia incidence ~ 1 + ID + apnoea type * apnoea duration". The dataset used in this model included 24 data points from 4 studies. The apnoea types were classified into 6 categories: central apnoea (n = 10), obstructive apnoea (n = 3), mixed central apnoea (n = 3), mixed obstructive apnoea (n = 3), unspecified apnoea (n = 3), and any apnoea (n = 2). * indicates p < 0.05, ** indicates p < 0.01, *** indicates p < 0.001.

**Name**	**Estimate**	**Standard Error**	**t Statistic**	**Degree of Freedom**	**p Value**
(Intercept)	-19.00	19.23	-0.99	12	0.34
Apnoea type (central)	13.26	19.48	0.68	12	0.51
Apnoea type (obstructive)	-12.92	37.08	-0.35	12	0.73
Apnoea type (mixed central)	-17.93	36.48	-0.49	12	0.63
Apnoea type (mixed obstructive)	-25.70	41.55	-0.62	12	0.55
Apnoea type (any)	31.55	61.24	0.52	12	0.62
Apnoea duration	3.00	0.86	3.49	12	0.0045**
Apnoea type (central):Apnoea duration	-1.56	0.91	-1.72	12	0.11
Apnoea type (obstructive):Apnoea duration	1.41	2.09	0.68	12	0.51
Apnoea type (mixed central):Apnoea duration	1.64	1.89	0.87	12	0.40
Apnoea type (mixed obstructive):Apnoea duration	2.17	2.18	1	12	0.34
Apnoea type (any):Apnoea duration	-2.17	3.39	-0.64	12	0.53

Supplementary Table 7: Results of the statistical analysis investigating if apnoea type modulates the relationship between oxygen saturation and apnoea duration. Fixed effects coefficients from the linear mixed-effects model with the formula: "oxygen saturation fall ~ 1 + ID + apnoea type * apnoea duration". The dataset used in this model included 39 data points from 5 studies. The apnoea types were classified into 5 categories: central apnoea (n = 12), obstructive apnoea (n = 5), mixed apnoea (n = 5), central/mixed apnoea (n = 12), and unspecified apnoea (n = 5). * indicates $p < 0.05$, ** indicates $p < 0.01$, *** indicates $p < 0.001$.

Name	Estimate	Standard Error	t Statistic	Degree of Freedom	p Value
(Intercept)	-0.20	0.47	-0.42	29	0.68
Apnoea type (mixed)	-2.57	1.36	-1.90	29	0.068
Apnoea type (obstructive)	-4.49	1.91	-2.35	29	0.026*
Apnoea type (unspecified)	0.18	1.55	0.12	29	0.91
Apnoea type (central/mixed)	3.55	1.23	2.89	29	0.0071**
Apnoea duration	-0.53	0.01	-39.34	29	< 0.0001***
Apnoea type (mixed):Apnoea duration	0.14	0.04	3.67	29	0.00098***
Apnoea type (obstructive):Apnoea duration	0.15	0.06	2.55	29	0.016*
Apnoea type (unspecified):Apnoea duration	0.01	0.10	0.09	29	0.93
Apnoea type (central/mixed):Apnoea duration	-0.07	0.07	-1.12	29	0.27

Supplementary Table 8: Results of the statistical analysis investigating if postmenstrual age (PMA) and apnoea type modulates the relationship between oxygen saturation and apnoea duration. Fixed effects coefficients from the linear mixed-effects model with the formula: "oxygen saturation fall ~ 1 + ID + apnoea type * apnoea duration + PMA * apnoea duration". The dataset used in this model included 39 data points from 5 studies. The apnoea types were classified into 5 categories: central apnoea (n = 12), obstructive apnoea (n = 5), mixed apnoea (n = 5), central/mixed apnoea (n = 12), and unspecified apnoea (n = 5). * indicates $p < 0.05$, ** indicates $p < 0.01$, *** indicates $p < 0.001$.

**Name**	**Estimate**	**Standard Error**	**t Statistic**	**Degree of Freedom**	**p Value**
(Intercept)	22.44	15.78	1.42	27	0.17
PMA	-0.78	0.54	-1.45	27	0.16
Apnoea type (mixed)	-0.75	1.67	-0.45	27	0.65
Apnoea type (obstructive)	-2.68	2.07	-1.29	27	0.21
Apnoea type (unspecified)	5.89	4.06	1.45	27	0.16
Apnoea type (central/mixed)	7.76	2.99	2.60	27	0.015*
Apnoea duration	-1.68	0.50	-3.34	27	0.0025**
PMA:Apnoea duration	0.04	0.02	2.29	27	0.030*
Apnoea type (mixed):Apnoea duration	0.06	0.05	1.17	27	0.25
Apnoea type (obstructive):Apnoea duration	0.07	0.07	1.03	27	0.31
Apnoea type (unspecified):Apnoea duration	-0.27	0.15	-1.80	27	0.083
Apnoea type (central/mixed):Apnoea duration	-0.28	0.11	-2.58	27	0.016*

Supplementary Table 9: Results of the statistical analysis investigating if postmenstrual age (PMA) modulates the relationship between heart rate drops and apnoea duration. Fixed effects coefficients from the linear mixed-effects model with the formula: “heart rate fall ~ 1 + ID + apnoea type * apnoea duration + PMA * apnoea duration”. The dataset used in this model included 23 data points from 5 studies. * indicates $p < 0.05$, ** indicates $p < 0.01$, *** indicates $p < 0.001$.

Name	Estimate	Standard Error	t Statistic	Degree of Freedom	pValue
(Intercept)	6.86	55.09	0.12	19	0.90
PMA	-0.13	1.52	-0.08	19	0.93
Apnoea duration	-2.70	2.48	-1.09	19	0.29
PMA:Apnoea duration	0.03	0.07	0.46	19	0.65

Supplementary Table 10(A): JBI Critical Appraisal Checklist for the included cross-sectional studies (Part A).

**Variables assessed**	**Daily et al. (1969)**	**Fenichel et al. (1980)**	**Werthammer et al. (1983)**	**Muttitt et al. (1988)**	**Curzi-Dascalova et al. (1989)**	**Suichies et al. (1989)**	**Hodgman et al. (1990)**	**Mathew et al. (1991)**	**Abdulhamid et al. (1992)**	**Finer et al. (1992)**	**Poets et al. (1993)**	**Poets et al. (1995)**
Were the criteria for inclusion in the sample clearly defined?	No	No	No	No	No	No	Yes	No	No	No	No	Yes
Were the study subjects and the setting described in detail?	Yes	No	No	Yes	Unclear	Unclear	Yes	Yes	Unclear	Unclear	Unclear	Unclear
Was the exposure measured in a valid and reliable way?	Yes	Unclear	Yes	Yes	Unclear	Unclear	Unclear	Yes	Yes	Unclear	Unclear	Unclear
Were objective, standard criteria used for measurement of the condition?	Yes	Yes	Yes	Yes	Yes	Yes	Yes	Yes	Yes	Yes	Yes	Yes
Were confounding factors identified?	No	No	No	Yes	Yes	No	No	No	No	No	No	Yes
Were strategies to deal with confounding factors stated?	N/A	N/A	N/A	Yes	Yes	N/A	N/A	N/A	N/A	N/A	N/A	No
Were the outcomes measured in a valid and reliable way?	Yes	No	Yes	Unclear	Yes	Yes	Yes	Yes	Yes	Yes	Yes	Yes
Was appropriate statistical analysis used?	N/A	N/A	N/A	Yes	N/A	Yes	Unclear	N/A	Unclear	Yes	N/A	Yes

Supplementary Table 10(B): JBI Critical Appraisal Checklist for the included cross-sectional studies (Part B).

**Variables assessed**	**Jenni et al. (1996)**	**Adams et al. (1997)**	**Carbone et al. (1999)**	**Curzi-Dascalova et al. (2000)**	**Nock et al. (2004)**	**Cardot et al. (2007)**	**Tourneux et al. (2008)**	**Beck et.al (2011)**	**Mohr et al. (2015)**	**Seppa-Moilanen et al. (2021)**	**Varisco et al. (2022)**
Were the criteria for inclusion in the sample clearly defined?	No	No	No	Yes	Yes	No	No	Yes	No	Yes	Yes
Were the study subjects and the setting described in detail?	Yes	Yes	No	Unclear	Unclear	Unclear	Unclear	Unclear	Yes	Unclear	Unclear
Was the exposure measured in a valid and reliable way?	Unclear	Yes	Unclear	Unclear	Unclear	Unclear	Yes	Yes	Yes	Yes	Yes
Were objective, standard criteria used for measurement of the condition?	Yes	Yes	Yes	Yes	Yes	Yes	Yes	Yes	Yes	Yes	Yes
Were confounding factors identified?	No	No	No	Yes	No	Yes	No	Yes	No	Yes	Yes
Were strategies to deal with confounding factors stated?	N/A	N/A	N/A	Yes	N/A	No	N/A	No	N/A	Yes	No
Were the outcomes measured in a valid and reliable way?	Yes	Yes	Yes	Yes	Yes	Yes	Yes	Yes	Yes	Yes	Yes
Was appropriate statistical analysis used?	Yes	Unclear	Unclear	Yes	Yes	Yes	Yes	Yes	Yes	Yes	N/A

Supplementary Table 11: JBI Critical Appraisal Checklist for the included cohort studies.

**Variables assessed**	**Gabriel et al. (1976)**	**Storrs (1977)**	**Vyas et al. (1981)**	**Southall et al. (1983)**	**Henderson-Smart et al. (1986)**	**Mathew (1988)**	**Waggener et al. (1989)**	**Upton et al. (1991)**	**Spear et al. (1992)**	**Upton et al. (1992) (A)**	**Upton et al. (1992) (B)**	**Urlesberger et al. (1999)**	**Pichler et al. (2003)**	**Fathabadi et al. (2017)**	**Marshall et al. (2019)**
Were the two groups similar and recruited from the same population?	N/A	N/A	N/A	Yes	N/A	N/A	N/A	N/A	No	N/A	N/A	Yes	Unclear	N/A	N/A
Were the exposures measured similarly to assign people to both exposed and unexposed groups?	N/A	N/A	N/A	Yes	N/A	N/A	N/A	N/A	Yes	N/A	N/A	No	Yes	N/A	N/A
Was the exposure measured in a valid and reliable way?	Unclear	Unclear	Yes	Yes	Yes	Yes	Yes	Unclear	Yes	Unclear	Yes	Unclear	Yes	Yes	Yes
Were confounding factors identified?	No	No	Yes	No	Yes	No	No	Yes	Yes	Yes	Yes	No	No	No	Yes
Were strategies to deal with confounding factors stated?	N/A	No	Yes	N/A	No	N/A	N/A	No	Yes	Yes	Yes	N/A	N/A	N/A	Yes
Were the groups/participa nts free of the outcome at the start of the study (or at the moment of exposure)?	Unclear	Unclear	No	No	Unclear	Yes	Unclear	Yes	Unclear	Unclear	Unclear	No	Unclear	Unclear	Unclear
Were the outcomes measured in a valid and reliable way?	Unclear	Yes	Yes	Yes	Yes	Yes	Yes	Yes	Yes	Unclear	Yes	Yes	Yes	No	Yes
Was the follow up time reported and sufficient to be long enough	Unclear	Unclear	Unclear	Unclear	Unclear	Yes	Unclear	Unclear	Unclear	Unclear	Unclear	Unclear	Unclear	Unclear	Unclear

for outcomes to occur?															
Was follow up complete, and if not, were the reasons to loss to follow up described and explored?	Unclear	Unclear	Unclear	Unclear	Unclear	Yes	Unclear	Unclear	Unclear	N/A	Unclear	Unclear	Unclear	Unclear	Unclear
Were strategies to address incomplete follow up utilized?	No	No	No	No	No	No	No	No	No	N/A	No	No	No	No	No
Was appropriate statistical analysis used?	N/A	Unclear	N/A	N/A	Yes	Yes	Yes	Yes	No	Yes	Yes	Yes	Yes	Yes	Yes

Supplementary Table 12: JBI Critical Appraisal Checklist for the included case control studies.

**Variables assessed**	**Poets & Southall (1991)**	**Elder et al. (2011)**
Were the groups comparable other than the presence of disease in cases or the absence of disease in controls?	Unclear	No
Were cases and controls matched appropriately?	Yes	Yes
Were the same criteria used for identification of cases and controls?	Unclear	Yes
Was exposure measured in a standard, valid and reliable way?	Yes	Unclear
Was exposure measured in the same way for cases and controls?	Yes	Yes
Were confounding factors identified?	No	No
Were strategies to deal with confounding factors stated?	N/A	N/A
Were outcomes assessed in a standard, valid and reliable way for cases and controls?	Yes	Yes
Was the exposure period of interest long enough to be meaningful?	Yes	Yes
Was appropriate statistical analysis used?	Yes	N/A

Supplementary Table 13: JBI Critical Appraisal Checklist for the included case report study.

**Variables assessed**	**Falsaperla et al. (2019)**
Were patient's demographic characteristics clearly described?	Yes
Was the patient's history clearly described and presented as a timeline?	Yes
Was the current clinical condition of the patient on presentation clearly described?	Yes
Were diagnostic tests or assessment methods and the results clearly described?	No
Was the intervention(s) or treatment procedure(s) clearly described?	Unclear
Was the post-intervention clinical condition clearly described?	Unclear
Were adverse events (harms) or unanticipated events identified and described?	No
Does the case report provide takeaway lessons?	Yes

Supplementary Table 14: Overall risk of bias assessment.

Study	Checklist	Internal Validity 'No' Responses	Overall Risk of Bias
Gabriel et al. (1976)	Cohort	2	High
Storrs (1977)	Cohort	3	High
Vyas et al. (1981)	Cohort	2	High
Southall et al. (1983)	Cohort	3	High
Henderson-Smart et al. (1986)	Cohort	2	High
Mathew (1988)	Cohort	2	High
Waggener et al. (1989)	Cohort	2	High
Upton et al. (1991)	Cohort	2	High
Spear et al. (1992)	Cohort	2	High
Upton et al. (1992) (A)	Cohort	0	Low
Upton et al. (1992) (B)	Cohort	1	Moderate
Urlesberger et al. (1999)	Cohort	4	High
Pichler et al. (2003)	Cohort	2	High
Fathabadi et al. (2017)	Cohort	3	High
Marshall et al. (2019)	Cohort	1	Moderate
Poets & Southall (1991)	Case-Control	1	Moderate
Elder et al. (2011)	Case-Control	2	High
Daily et al. (1969)	Cross-Sectional	1	Moderate
Fenichel et al. (1980)	Cross-Sectional	2	High
Werthammer et al. (1983)	Cross-Sectional	1	Moderate
Muttitt et al. (1988)	Cross-Sectional	0	Low
Curzi-Dascalova et al. (1989)	Cross-Sectional	0	Low
Suichies et al. (1989)	Cross-Sectional	1	Moderate
Hodgman et al. (1990)	Cross-Sectional	1	Moderate
Mathew et al. (1991)	Cross-Sectional	1	Moderate
Abdulhamid et al. (1992)	Cross-Sectional	1	Moderate
Finer et al. (1992)	Cross-Sectional	1	Moderate
Poets et al. (1993)	Cross-Sectional	1	Moderate
Poets et al. (1995)	Cross-Sectional	1	Moderate

Jenni et al. (1996)	Cross-Sectional	1	Moderate
Adams et al. (1997)	Cross-Sectional	1	Moderate
Carbone et al. (1999)	Cross-Sectional	1	Moderate
Curzi-Dascalova et al. (2000)	Cross-Sectional	0	Low
Nock et al. (2004)	Cross-Sectional	1	Moderate
Cardot et al. (2007)	Cross-Sectional	1	Moderate
Tourneux et al. (2008)	Cross-Sectional	1	Moderate
Beck et.al (2011)	Cross-Sectional	1	Moderate
Mohr et al. (2015)	Cross-Sectional	1	Moderate
Seppa-Moilanen et al. (2021)	Cross-Sectional	0	Low
Varisco et al. (2022)	Cross-Sectional	1	Moderate
Falsaperla et al. (2019)	Case Report	2	High
